# Rotator side chains trigger cooperative transition for shape and function memory effect in organic semiconductors

**DOI:** 10.1038/s41467-017-02607-9

**Published:** 2018-01-18

**Authors:** Hyunjoong Chung, Dmytro Dudenko, Fengjiao Zhang, Gabriele D’Avino, Christian Ruzié, Audrey Richard, Guillaume Schweicher, Jérôme Cornil, David Beljonne, Yves Geerts, Ying Diao

**Affiliations:** 1 Department of Chemical and Biomolecular Engineering, University of Illinois at Urbana−Champaign, 600 South Mathews Avenue, Urbana, IL 61801 USA; 2 Laboratory for Chemistry of Novel Materials, University of Mons, Place du Parc, 20, B-7000 Mons, Belgium; 3 Institut Néel, CNRS and Grenoble Alpes University, 25 rue des Martyrs, 38042 Grenoble, France; 4 Laboratoire de Chimie des Polymères Faculté des Sciences, Université Libre de Bruxelles (ULB), CP206/1, Boulevard du Triomphe, 1050 Brussels, Belgium; 5 Optoelectronics Group Cavendish Laboratory, University of Cambridge, JJ Thomson Avenue, Cambridge, CB3 0HE UK

## Abstract

Martensitic transition is a solid-state phase transition involving cooperative movement of atoms, mostly studied in metallurgy. The main characteristics are low transition barrier, ultrafast kinetics, and structural reversibility. They are rarely observed in molecular crystals, and hence the origin and mechanism are largely unexplored. Here we report the discovery of martensitic transition in single crystals of two different organic semiconductors. In situ microscopy, single-crystal X-ray diffraction, Raman and nuclear magnetic resonance spectroscopy, and molecular simulations combined indicate that the rotating bulky side chains trigger cooperative transition. Cooperativity enables shape memory effect in single crystals and function memory effect in thin film transistors. We establish a molecular design rule to trigger martensitic transition in organic semiconductors, showing promise for designing next-generation smart multifunctional materials.

## Abstract (web summary)

Martensitic transition is commonly seen in steel and shape memory alloys but rarely in organic materials. Chung et al. discover martensitic transitions in organic electronics and utilize it in designing field-effect transistors, leading to shape memory effect that in return modifies charge transport properties.

## Introduction

Cooperativity has long been used by living systems to circumvent energetic and entropic barriers to yield highly efficient molecular processes^1^. One type of cooperative structure transition is martensitic transition, which involves a simultaneous, concerted displacement of molecules in a crystalline material^2,3^. Materials exhibiting martensitic transition have high application potential as tiny machines^4^, amplifying subnanometer motion to macroscopic behavior. Martensitic transition has acquired much attention in the research community for its low transition barrier, ultrafast kinetics, and structural reversibility^5^. A key characteristic is the shape memory effect^6,7^, whose applications range from medical and drug delivery devices, actuators, and sensors in the automotive and aerospace industry^5,8^.

Martensitic transition has been extensively studied in metallic crystals, alloys, and ceramics, but only a few analogous studies have been done in organic systems. One is the discovery of superelasticity in terephthalamide crystal by Takamizawa et al.^9,10^. They demonstrated a more precise controllability of superelasticity than in metal alloys. Another example is thermosalient crystals, also known as ‘jumping crystals’^11,12^, which exhibit near-instantaneous martensitic transition and display the shape memory effect. These studies are mainly phenomenological, and the molecular mechanism and origin are still in question. Anwar et al.^13^ used molecular dynamics simulation in DL-norleucine crystals to report concerted bilayer displacement during martensitic transition. On the other hand, Mnyukh^14^ argues that martensitic transition occurs molecule by molecule without concerted molecular movement at the interface between the parent and daughter phase as epitaxial nucleation and growth.

Previous studies^15–19^ have linked cooperative transition in organic systems to changes in molecular orientation. A recent review by Sato^15^ comprehensively summarizes that the changes in molecular orientation often originate from displacement, rotation, and/or an order-to-disorder transition of molecules in molecular crystals, leading to switchable physical properties including the shape memory effect. Yao et al.^16^ reported that a 90° molecular rotation of the oxalate molecules in a crystalline Ni complex caused a cooperative interaction of the molecules to show a contraction and expansion of the crystal. Su et al.^18^ showed that an approximately 100° rotation of the *n*-butyl group induced a cooperative first-order-phase transition in single crystals of a cobalt(II) complex. However, these studies are limited to one system, and generalizable molecular design rules have not been reported for inducing martensitic transition and switchable physical properties in molecular crystals.

Furthermore, martensitic transition has not been observed in organic semiconductor systems so far. Organic semiconductors underpin the rapidly advancing printed electronics technology that promises flexible, light-weight, biointegrated electronics at low cost and high throughput in forms unimagined before^20–25^. Merging these two research areas by coupling electronic switching and sensing mechanisms with the shape memory effect can open new opportunities in creating shape memory electronics. In addition, uncovering molecular design rules to induce martensitic transition can lead to new strategies to access high-performing polymorphs while maintaining structural integrity^26–28^. Toward these design objectives, understanding the origin and mechanism of martensitic transition in molecular crystals is a prerequisite.

In this study, we establish bulky side chain rotation as a new molecular mechanism for triggering martensitic transitions in molecular crystals. We study this phenomenon using single crystals and thin films of two high-performing p-type organic semiconductors, ditert-butyl [1] benzothieno[3,2-b][1]1benzothiophene (ditBu-BTBT)^29^ and 6,13-bis(triisopropylsilylethnyl) pentacene (TIPS-pentacene)^27,30–33^. Using in situ polarized optical microscopy (POM), we observe a solid-state-phase transition that drastically differs from the commonly known nucleation and growth mechanism. From single-crystal X-ray diffraction (SCXRD), Raman and nuclear magnetic resonance (NMR) spectroscopy, and molecular dynamics simulations, we infer that the rotation of the bulky *tert*-butyl side chains at elevated temperature triggers martensitic transition. We then prove generality using TIPS-pentacene single crystals, which exhibit a pronounced reversible shape memory effect characteristic of martensitic transitions. We further demonstrate in thin film transistor devices of both systems that cooperative transition enables large magnitude, reversible switching of charge carrier mobility over multiple cycles, which we term as ‘function memory effect’. Herein, we explicitly define ‘memory effect’ as abrupt, complex, reversible change in properties (i.e., shape, electronic/optical properties) triggered by cooperative structural transitions. As shown in this work, such ‘memory effect’ is fundamentally different from simple temperature-dependent property change, in that the magnitude of change is several fold to orders of magnitude larger over the same temperature range, and that the effect can be complex, exhibiting negative thermal expansion along certain unit cell axis and mobility change opposite to temperature-dependent behavior.

## Results

### Cooperative transition in ditBu-BTBT single crystals

A single crystal is an ideal platform for studying solid-state-phase transitions^34,35^ and revealing structure-charge transport relationship in organic electronic devices^36–41^. The absence of grain boundaries makes direct observation of transformation kinetics much easier and reproducible. We developed a simple dropcasting method for fabricating single crystals in solution ranging from 50 to 400 µm in size with thickness of several microns (Methods section).

We employed POM equipped with a hot stage for in situ observation of ditBu-BTBT single crystals. Thirty single-crystal samples were subject to heating and cooling rate of 2 K min^−1^. We observed single-crystal to single-crystal transition^35,42,43^ at 345 K on average during heating and 331 K during cooling. We observed a wide range of transition temperatures: for the first heating cycle, they ranged between 342 and 350 K (Supplementary Table 1). They were comparable to single-crystal differential scanning calorimetry (DSC) data using 2 K min^−1^ heating rate, yielding 347.1 and 346 K for heating and cooling transition temperatures, respectively (Supplementary Fig. 1). The difference in transition temperature hysteresis between in situ POM and DSC may be attributed to the difference in fabrication process (solution versus physical vapor transport) or instrument stage (glass slide versus aluminum crucible). Nonetheless, the transition temperature hysteresis observed in both POM and DSC suggests that the transition is activated and first order.

**Fig. 1:**
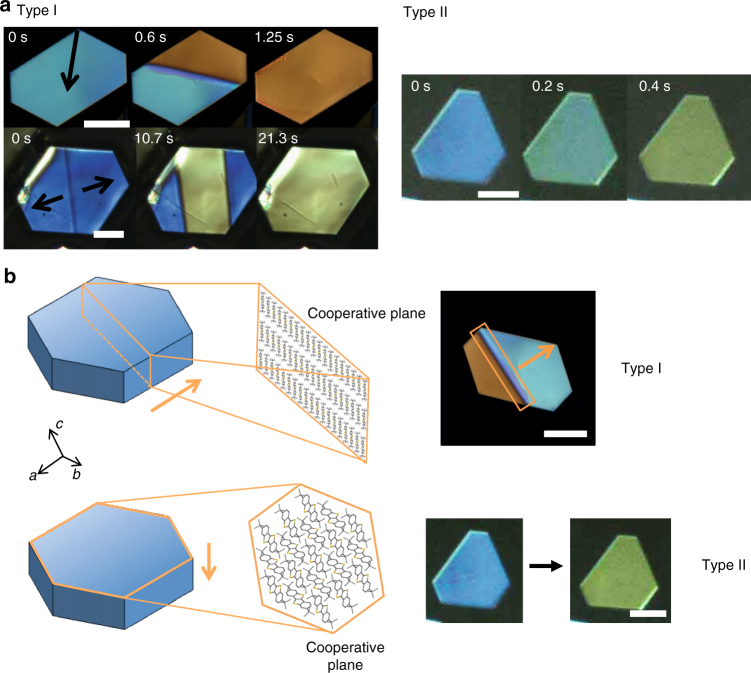
Martensitic transition in ditBu-BTBT single crystals. **a** POM images of ditbu-BTBT single crystals displaying Types I and II transition from LT (blue) to HT (yellow). The arrows in Type I panel indicate direction of movement of the phase boundary line between LT and HT forms. Top and bottom scale bars correspond to 100 and 25 µm, respectively. Type II transition exhibits a uniform change in birefringence across the entire crystal surface. Scale bar corresponds to 25 µm. **b** Schematic of the cooperative molecular planes (enclosed molecules) involved in Type I and Type II transitions. The two types are differentiated in terms of the direction of phase boundary propagation. Top and bottom scale bars correspond to 100 and 25 µm, respectively

Surprisingly, we observed a distinct phase boundary line sweep across all crystal samples on the order of seconds or less. Birefringence changed between blue and yellow as the crystal was heated, and the process was reversible upon cooling (Fig. 1a, Type I, Supplementary Movies 1 and 2). The difference in birefringence indicates change in the refractive index ellipsoid and the effective electric polarizability of the crystal. We attribute this to the structural change of the crystal, and assign the ‘blue’ and ‘yellow’ phases as the low-temperature (LT) and the high-temperature (HT) polymorphs, respectively. In addition, several single-crystal samples exhibited a uniform birefringence color change on the entire crystal surface without the appearance of a phase boundary line (Fig. 1a, Type II, Supplementary Movie 3). We denote these two transition behaviors as Type I and Type II, respectively. Regardless of Type I or II transition, we observed a small, reversible shape change of the crystal upon transition.

There were distinct differences between our observation and the nucleation and growth transition^14,34,35,42^. First, by nucleation and growth, the process initiates at a point in the crystal and propagate outward at velocities inversely proportional to the interfacial energies of the corresponding facets. In contrast, we observed a concerted movement of the phase boundary. Second, due to the stochastic nature of nucleation, the orientation of the daughter phase is not bound to that of the parent phase^42^, whereas we observed a close registration of the daughter with the parent phase. Third, the nucleation and growth process in similar-sized crystals is usually very slow, spanning hours to days, as opposed to seconds or less observed in this study. For instance, in two previous studies which reported nucleation and growth mechanism^34,42^, the growth rates in single crystals were estimated to be 0.03 and 0.007 µm s^−1^. In contrast, we observed an average propagation speed of 188 µm s^−1^ for Type I transition with the highest speed reaching 641 µm s^−1^. We attribute the much higher transition speeds to molecular cooperativity. Furthermore, most of the reported martensitic transitions are activated and first order^2^, in agreement with our results. All these observations in ditBu-BTBT conform to the characteristics of martensitic transition.

Next we infer how molecular cooperativity, which forms the basis of a martensitic transition, leads to Type I and II transitions. Bravais–Friedel–Donnay–Harker (BFDH) morphology calculation indicates that the molecules stack in layers out of plane along the *c*-axis (Supplementary Fig. 2). In Type I, a layer of molecules at the cross-section along the phase boundary line propagate in-plane across the crystal. In Type II, an entire layer of molecules parallel to the substrate propagate out of plane (Fig. 1b). We reason that Type II requires significantly more molecules to transform concertedly than the case of Type I, as the width of the crystal is usually much greater than the thickness. Not surprisingly, we only observed 2 cases of Type II compared to 28 cases of Type I. Samples that exhibit Type I have a crystal width at least 50 times larger than the thickness. Occasionally, we observed both types of transitions in small crystals that are nearly 50 wide and 20 µm thick. We deduce that the preferred direction of propagation is determined by the total number of molecules involved in the propagation plane. The larger the width-to-thickness ratio of the crystal, the more likely the crystal will exhibit Type I, as it involves less molecules for cooperative propagation and thus has a lower free energy barrier to overcome.

### Molecular origin of cooperative transition in ditBu-BTBT

**Fig. 2:**
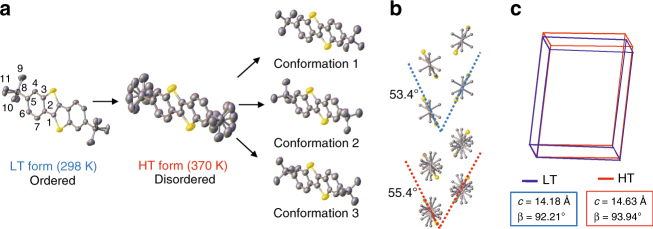
Molecular structure differences between the LT and the HT polymorphs in ditBu-BTBT. **a** Order-to-disorder transition of the side chain and the three conformations determined from SCXRD. The carbon atoms in the asymmetric unit are labeled. **b** Change in herringbone dihedral angle between the LT and HT polymorphs. **c** Comparison of unit cells before and after the transition. The unit cell parameters with the most pronounced changes are labeled. Hydrogen atoms are omitted for clarity

We elucidate the molecular origin of martensitic transition observed in ditBu-BTBT single crystals using a combination of SCXRD, NMR and Raman spectroscopy. SCXRD experiments were conducted on the LT and HT forms of the ditBu-BTBT single crystal at 298 and 370 K, respectively (Supplementary Table 2). Thanks to martensitic transition which preserves the structural integrity, we obtained both LT and HT crystal structures from the same single crystal by thermally triggering the transition in the diffractometer. The most pronounced difference is that the HT form exhibits side chain rotational disorder, whereas the LT form does not. In other words, the side chains in the LT form reside in a stable conformation until thermal energy induces an order-to-disorder transition, causing the side chains in the HT form to rotate while the backbone remains fixed. From the crystal structure of the HT form, we determined that the *tert*-butyl side chains are toggling among three conformations with distinct side chain rotation angles, each with the chemical occupancy probability of 0.41, 0.37, and 0.22, respectively (Fig. 2a).

The side chain order-to-disorder transition is accompanied by small but perceivable changes in molecular packing and unit cell structures. From the LT to the HT form, the herringbone packing motif is preserved, with a 2° change in the dihedral angle of the herringbone pair (Fig.  2b), a difference significant enough to alter the charge transfer integrals and electronic properties discussed later. Both LT and HT polymorphs belong to monoclinic system with P2_1_/c space group; the *c* axis and β-angle show the largest differences among all unit cell parameters (Fig.  2c). The unit cell changes, albeit small, are not due to thermal expansion. We ruled out this possibility based on the observation that the unit cell parameters went through a much more significant anisotropic change from 298 to 370 K (martensitic transition) compared to from 370 to 400 K (thermal expansion only) (Supplementary Table 3). These small structural changes of the unit cell translated to macroscopic alteration of the crystal shape during transition, as observed from in situ POM (Supplementary Fig. 3). These results led us to hypothesize that the order-to-disorder transition of the bulky side chains causes rearrangement of molecular packing, deforms the unit cell, and triggers the cooperative transition of the entire crystal.

**Fig. 3:**
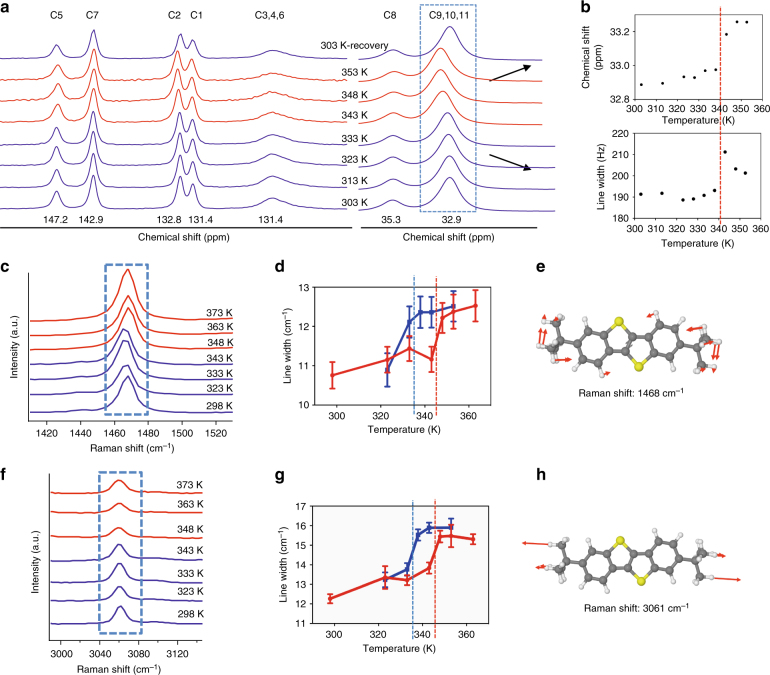
Experimental evidence showing side chain disorder in ditBu-BTBT. **a** Variable temperature NMR spectra of ditBu-BTBT powder sample. Blue and red spectra correspond to LT and HT forms, respectively. The peak that corresponds to 32.9 ppm was identified as the side chain carbons, C9, C10, and C11. **b** Both the chemical shift position and the linewidth of the side chain carbons show a clear increase near the predicted transition temperature from DSC (red dashed line). **c** Variable temperature Raman spectra for Raman shift 1468 cm^−1^. **d** Plot of linewidth as a function of temperature for Raman shift 1468 cm^−1^. **e** DFT predicted vibrational mode showing C-H bond bending in the side chains. **f** Variable temperature Raman spectra for Raman shift 3061 cm^−1^. **g** Plot of linewidth as a function of temperature for Raman shift 3061 cm^−1^. **h** DFT prediction showing C-H asymmetric stretching in the side chains. Blue and red spectra correspond to LT and HT forms, respectively. A clear increase in the linewidth is observed at the predicted transition temperatures for both Raman shifts (red dashed line: onset transition temperature during heating, blue dashed line: onset transition temperature during cooling). The error bars are calculated from the Gaussian curve fitting of the Raman spectra

In addition to SCXRD, solid-state NMR spectroscopy is an excellent technique to observe the local molecular changes that occur during a phase transition^18,19,44^. For this system, we focused on the carbons of the tBu side chains (C9, C10, and C11), which appeared on the NMR spectrum near 32.9 ppm We conducted variable temperature ^13^C cross-polarization magic angle spinning solid-state NMR spectroscopy for a ditBu-BTBT powder sample. The temperature was varied incrementally to cover the LT–HT transition temperature, 343.7 K, predicted from DSC (Fig. 3a). Interestingly, the peak associated with the tBu side chains showed clear chemical shift of ~0.3 ppm as well as an abrupt increase in the linewidth of 20 Hz near 343 K (Fig. 3b). When cooled back, both the chemical shift and the linewidth returned to original values, proving reversibility (Fig. 3a). Chemical shifts of NMR peaks were associated with molecular structural changes in previous studies that focused on polymorph transitions^19,44^. It has been reported previously^45,46^ that increase in linewidths of an NMR peak reflects spatial disorder in the molecule. Thus, the NMR results indicate that the tBu side chains start showing rotational disorder as the polymorphic phase transition takes place, agreeing with SCXRD results. This result offers evidence that the side chain conformational disorder triggers cooperative transition.

We next used variable temperature Raman spectroscopy to validate the local molecular changes in the tBu side chains before and after transition. This technique has been used to study the molecular origin of phase transition in small molecules such as amino acids^47–50^ and liquid crystals^51^. With the microscope and camera attached to the instrument, we could observe the slight crystal shape changes and thus pinpoint the exact temperature when cooperative transition was taking place.

Single crystals of ditBu-BTBT yielded sharp Raman peaks without visible beam damage over the course of the thermal cycle. We varied the stage temperature and noted a clear change in the shape of the crystal between 343 and 348 K on heating, and between 338 to 333 K on cooling. Around these transition temperatures, we observed abrupt increases in the linewidths of 1468 (Fig. 3c, d) and 3061 cm^−1^ Raman bands (Fig. 3f, g). We conducted density functional theory (DFT) calculations and validated that the peak at 1468 cm^−1^ corresponds to the carbon–hydrogen bond bending in the side chains (Fig. 3e), and the 3061 cm^−1^ peak to the carbon–hydrogen bond asymmetric stretching in the side chains (Fig. 3h). We attribute the abrupt increase in peak widths for 1468 and 3061 cm^−1^ Raman bands upon LT to HT transition to increased side chain disorder. Our inference is consistent with studies which showed that an abrupt increase in linewidth indicates onset of disorder in chemical bonds corresponding to the specific Raman shift^47,48,51^.

The SCXRD, NMR, and Raman studies provided solid experimental evidence that the order-to-disorder transition of the side chains is the molecular origin of cooperative transition in the ditBu-BTBT system.

### Understanding cooperativity in side chain rotation

**Fig. 4:**
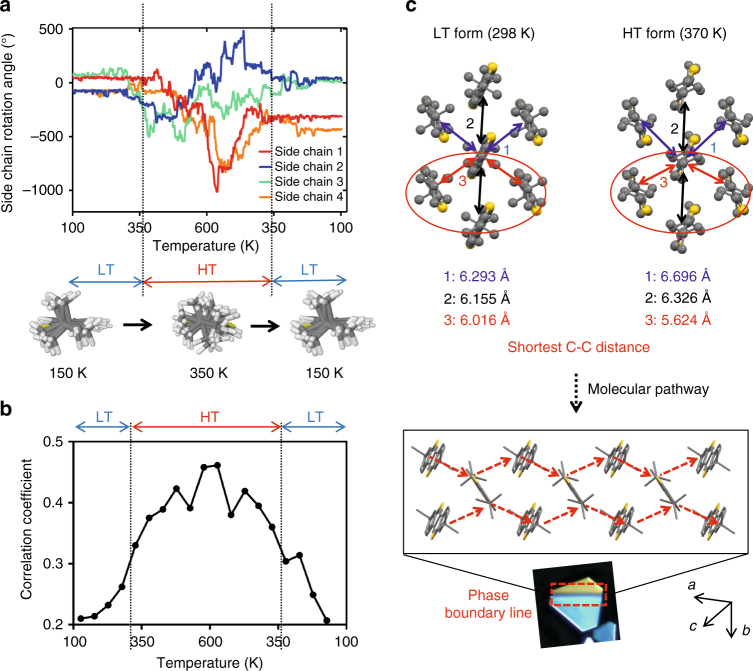
Bulky side chain rotation triggers molecular cooperativity and martensitic transition. **a** Rotation angles of four side chains as a function of temperature from 100→600→100 K. The side chain conformations at 150 and 350 K are illustrated beneath, created by superimposing all conformations observed at the corresponding temperatures. The result indicates freely rotating side chains in the HT form. Vertical dashed lines indicate onset of fluctuations in the side chain rotation upon heating and cooling, which match closely with the observed and measured transition temperature. **b** Temperature dependence of the average correlation coefficient between rotations of neighboring ditBu side chains. **c** Inferred molecular pathway for forming the phase boundary line shown in Fig. 1a Type I. The center carbon distances of the tert-butyl groups between molecular pairs were calculated from the crystal structures for both the LT and HT forms. The red arrows indicate the shortest intermolecular distance in each form. Extension along the shortest intermolecular distance for both side chains on a single molecule constitutes a possible molecular pathway that forms the phase boundary line observed in Type I transition

To evaluate our hypothesis that molecular cooperativity underlies the polymorph transition observed, we investigated the extent of correlation in the rotational motion of neighboring side chains via molecular dynamic simulations and statistical analysis. We first conducted molecular simulations of the side chain order-to-disorder transition as a function of temperature. As shown in Fig. 4a, the side chain rotation angles of four arbitrarily chosen ditBu-BTBT molecules were monitored as the simulation cell was first heated from 100 to 600 K and then cooled down to 100 K. From 100 to 350 K, the side chains show a low amplitude motion around their potential minima. Starting at 350 K, the rotational motions become more intense, gaining sufficient energy to overcome three-fold potential barriers and result in free rotational motion. In other words, the ordered back-and-forth rotation intensifies into a disordered spinning wheel motion above 350 K. The side chains stabilize again when cooled below 330 K. The simulated order-to-disorder transition temperatures of 350 K (heating) and 330 K (cooling) match closely with the experimentally observed LT/HT transition temperatures of 345 K (heating) and 331 K (cooling). This result supports our hypothesis that the onset of side chain rotation triggers the polymorphic transition between LT and HT forms.

To investigate the collective nature of neighboring side chains as they become disordered, we performed additional statistical analysis on the MD simulations from Fig. 4a. To quantify the mutual dependence of the rotational dynamics of neighboring side chains, we calculated the average correlation coefficient, *R*. It ranges between 0 and 1 for completely uncorrelated and correlated motions, respectively (see Methods). The temperature dependence of the correlation coefficient, shown in Fig. 4b, reveals that the rotational motion is weakly correlated in the LT form with *R* of ~0.2, where the side chains only perform small oscillations around their equilibrium conformation. In the HT form, the rotational motion is significantly correlated, as *R* reaches higher than 0.4. Interestingly, while *R* varies continuously across the transition, the steepest change occurs around the transition temperature as determined from in situ POM and DSC. A closer inspection of the data reveals a qualitatively different distribution of *R* for each pair of ditBu groups in the LT and HT phases (Supplementary Fig. 4). At 175 K, which corresponds to the LT form, the motion of the large majority of ditBu pairs presents very weak correlations, irrespectively on the thermal history of the sample. The distribution at 600 K, which corresponds to the HT form, is flat, with *R* almost spanning the entire range from 0 to 1. This denotes the coexistence of incoherent and highly collective motions of the side groups. It is possible that highly cooperative side rotation is localized in small molecular clusters which appear stochastically across the entire crystal. This analysis proves that the motion of side chains gains a collective, cooperative character, albeit stochastic, as they transition from the LT to the HT form.

The cooperative nature of the side chain rotation is consistent with the inference from the entropy change during transition. According to Boltzmann’s equation, *S* = *R*ln*N*, where *R* is the gas constant and *N* is the number of microstates in the system. Going from LT to HT, the number of microstates associated with the side chain rotational conformation is expected to increase; the extent of increase in *N* depends on the level of cooperativity. We estimated *N* from Δ*S* obtained from the DSC data. At the equilibrium transition temperature, ΔS = ΔH/T. We approximated the equilibrium transition temperature as the midpoint of the transition temperatures during the heating and cooling cycle in DSC (Supplementary Fig. 1). We calculated Δ*S* = 10.4 J mol^−1^ K^−1^ to yield *N* to be about 3.5 for HT, assuming *N* = 1 for LT. This result is in line with our inference based on the SCXRD and MD results discussed above, that there are three distinct side chain conformations that exhibit partial cooperativity. Correspondingly, the number of microstates in the HT phase is close, but higher than 3.

We next infer the intermolecular interactions responsible for the molecular cooperativity during transition. In both the LT and HT crystal structures, each ditBu-BTBT molecule has six nearest neighbors (Fig. 4c). The herringbone packing is mainly guided by the attractive quadrupole interactions stabilized by dispersive interactions. The attractive interactions are balanced by short-range repulsion, or volume exclusion interactions, between the adjacent *tert*-butyl groups. We hypothesize that the steric hindrance experienced during side chain rotation readjusts the herringbone packing between molecules, triggering cooperative transition. Therefore, we expect the side chain rotation to propagate along a path with the strongest repulsion, or the shortest intermolecular distances, to form the phase boundary line experimentally observed. Given that the *tert*-butyl group rotates around the central carbon, we determined the shortest distances between adjacent center carbons to be 6.02 and 5.62 Å for LT and HT forms, respectively, both along the same herringbone pair (red arrows in Fig. 4c). Extension of this route in both directions (red dashed arrows in Fig. 4c) constitutes the phase boundary line dissecting the crystals. We observed that the phase boundary line always appeared along the same sets of crystallographic planes for Type I transition (Supplementary Fig. 5), consistent with the molecular mechanism proposed. Interestingly, after the formation of the phase boundary line along the herringbone pairs, its propagation direction coincided with the π–π stacking direction which is along the second closest molecular pairs (black arrows in Fig. 4c).

### Testing generality of hypothesis in TIPS-pentacene

**Fig. 5:**
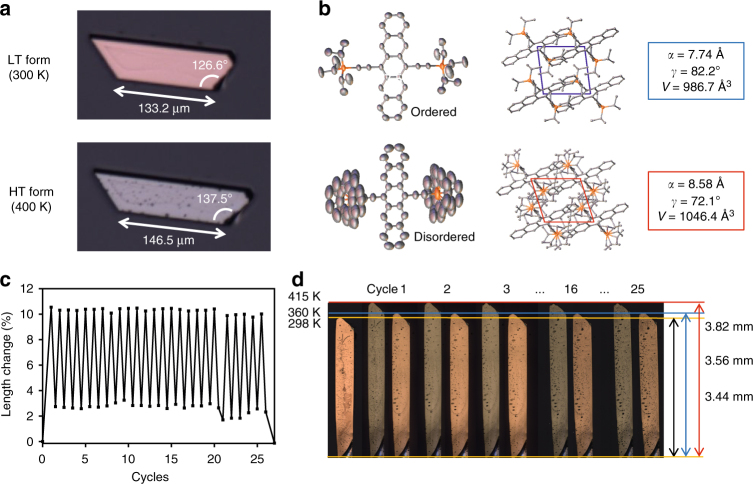
Martensitic transition in TIPS-pentacene single crystals showing the shape memory effect. **a** Length and angle changes before (300 K) and after (400 K) martensitic transition. **b** Order-to-disorder transition of the side chain. The molecular structure shown for the HT form is the overlap of three conformations, similar to the ditBu-BTBT system. The unit cell parameters that experienced the largest changes are shown. Hydrogen atoms are omitted for clarity. **c** Pronounced shape memory effect in a TIPS-pentacene single crystal displaying a length change of more than 10% for 26 cycles. Throughout the entire experiment, the sample showed precise and highly repeatable mechanical expansion and contraction. **d** POM image of the TIPS-pentacene single crystal tested for the shape memory effect. After 26 thermal cycles tested over a period of 8 weeks, structural integrity of the mm-sized TIPS-pentacene single crystal was preserved

To evaluate whether order-to-disorder transition of bulky side chains is potentially generalizable as a molecular design rule for cooperative transition, we examined the solid-state polymorphic transition^52,53^ in single crystals of TIPS-pentacene—a high-performing organic semiconductor. During in situ POM, we indeed observed a significant change in the crystal shape accompanied with uniform color change (Fig. 5a, Supplementary Movie 3) reminiscent of Type II transition in ditBu-BTBT single crystals (Fig. 1a). The long axes of five different single-crystal samples spanning microns to millimeters in size expanded by 10 to 12% upon heating to 400 K. The shape change was completely reversible upon cooling. The observed shape change appears to be a result of both a second-order transition and a first-order transition combined, and the most significant and abrupt shape change occurred during the first-order transition near 400 K (Supplementary Fig. 6). This agrees with powder DSC^27^, where a transition was predicted near 395 K. We infer that this first-order transition is martensitic in nature, given its reversibility, rapid transition kinetics, and that the orientation of the daughter phase is pre-defined by that of the parent phase. We note that shape memory effect induced by martensitic transitions is a phenomenon rarely observed in molecular crystals^10,12,16^. In fact, ditBu-BTBT also exhibits shape memory effect—we observed a small but sudden shape change during the martensitic transition, characterized by a perceivable alteration of the dihedral angles of the crystal. Such change was distinct from thermal expansion, as the crystal remained stationary during heating prior to the transition (Supplementary Fig. 3).

As expected, comparison between the single-crystal structures of the LT (300 K) and HT (400 K) forms revealed rotational disorder of triisopropyl side chains in the HT form, in contrast to static side chains in the LT form (Fig. 5b). The side chains of the HT form toggle among three conformations with chemical occupancy probabilities of 0.45, 0.33, and 0.22, respectively (Supplementary Fig. 7). Both LT and HT forms belong to the triclinic system with space group P$\bar 1$ and exhibit brick wall packing motifs (Fig. 5b). On the other hand, the unit cell parameters of the two forms differ significantly, with the largest differences found in the *a-* axis and the *γ*-angle (Fig. 5b, Supplementary Table 4). Comparing HT to LT unit cells, the *a-*axis increases by 10.8%, comparable to the extent of crystal elongation during transition. We deduce from simulated BFDH morphology that the *a*-axis is possibly parallel to the long axis of the crystal (Supplementary Fig. 8). Moreover, we infer that the side chain order-to-disorder transition propagates along all three axes of the unit cell by determining the shortest Si–Si distances, which are distances of the side chain rotation centers (Supplementary Fig. 9). This inference is consistent with the microscopy observation that the entire crystal experiences uniform color change during transition (Supplementary Movie 4). All these results consistently point to the conclusion that the martensitic transition and the pronounced shape memory effect observed is a macroscopic manifestation of the large crystal structure change induced by order-to-disorder transition of the bulky triisopropyl side chain of TIPS-pentacene.

We also tested the shape memory effect through multiple thermal cycles. Millimeter-sized single-crystal rods showed significant expansion and contraction along the long axis during transition (Fig. 5c). Over 26 thermal cycles and with tests scattered over a period of 8 weeks, the crystal sample displayed highly stable and repeatable shape memory effect when cycled between 360 and 415 K. The structural integrity of the crystal was preserved throughout the test (Fig. 5d). We attribute the remarkable durability of the crystals during shape change to the strong π–π interaction between the pentacene rings, which serves to lock the molecules in place during numerous expansion and contraction cycles. Studies on single crystals that show the shape memory effect^12^ or elasticity and flexibility^9,54,55^ commonly display strong π–π interaction between the aromatic rings. Such interaction is also flexible enough to allow variation in the π–π stacking distance or offset during phase transition. On rare occasions, we observed self-healing of cracks that appeared during cooling in the subsequent heating cycle (Supplementary Fig. 10, Supplementary Movie 5). The occurrence of both the shape memory effect and the self-healing effect has been studied recently in terepthalic acid by Karothu et al.^12^, where these phenomena were described as macroscopic manifestation of martensitic transition.

We validate that the rotating bulky isopropyl side chains of TIPS-pentacene triggers martensitic-type transition, inducing pronounced and durable shape memory effect. This concept is potentially general, as we have demonstrated the same molecular origin of cooperative transition in two different organic semiconductors. We believe this is a significant first step towards formulating a molecular design strategy for actively inducing martensitic transitions in molecular crystals, opening doors to potential applications of martensitic transition in yet unexplored organic functional materials.

### Impact of cooperative transition on electronic properties

We evaluated charge transport characteristics in organic field-effect transistors (OFETs) of both ditBu-BTBT and TIPS-pentacene to establish the polymorph-charge transport relationship and to explore potential electronic application utilizing martensitic transitions. We focus on thin film geometry for device studies given its direct relevance to practical applications. Thin film samples were prepared using meniscus-guided coating described in our earlier work^56^. Thickness of the thin films fabricated were ~30 and 40 nm for ditBu-BTBT and TIPS-pentacene, respectively.

We first confirmed that polymorphic transition occurs in ditBu-BTBT thin films in a similar fashion as in single crystals, probed using in situ grazing incidence X-ray diffraction (GIXD) (Supplementary Fig. 11). We observed the start of polymorphic transition near 343 K upon heating of the thin film, compared to 345 K for single crystals. The transition was reversible and the structure integrity of the thin film was preserved, confirming the cooperative nature of the polymorphic transition. We also determined the unit cell parameters before and after transition using the thin film refinement technique^27^, which closely matched those solved using single-crystal samples (Supplementary Table 3).

**Fig. 6:**
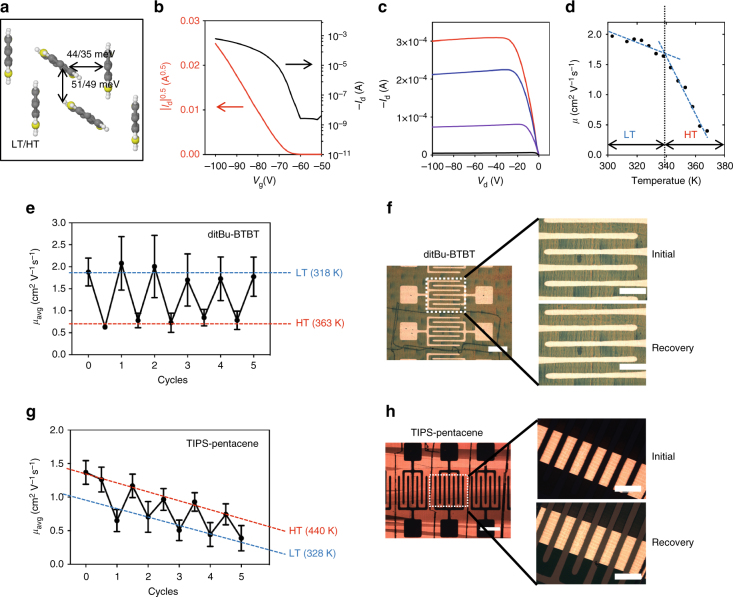
Electronic properties and function memory effect of thin film transistors. **a** Calculated charge transfer integrals in the LT and HT forms of ditBu-BTBT. **b** Transfer curve in the saturation regime (*V*_d_ = −100 V), **c** output curve and **d** temperature-dependent apparent mobility in the saturation regime for ditBu-BTBT OFETs. The dashed line indicates the transition temperature at the intersection of the two slopes. Data points are in 5 K increments **e** Reversible switching of apparent mobility of ditBu-BTBT thin film devices over five thermal cycles. Each mobility value is an average of four devices. Each error bar is calculated from the standard deviation of four devices. **f** Preservation of thin film morphology and device structure before and after a thermal cycle in ditBu-BTBT. Scale bar on the left corresponds to 500 µm, and the scale bars on the zoomed in images correspond to 100 µm. **g** Reversible switching of apparent mobility of TIPS-pentacene thin film devices over five thermal cycles. Each mobility value is an average of four devices. Each error bar is calculated from the standard deviation of four devices. **h** Preservation of thin film morphology and device structure before and after a thermal cycle in TIPS-pentacene. Scale bar on the left corresponds to 500 µm, and the scale bars on the zoomed in images correspond to 100 µm

To determine the extent to which the polymorphic transition alters the intrinsic charge transport property, we performed quantum-chemical calculations to compute the charge transfer integrals in the LT and HT forms (Fig. 6a). The transfer integrals decrease from LT to HT, from 44 to 35 and 51 to 49 meV for herringbone and π–π pairs, respectively. The results indicate a lower charge carrier mobility in the HT form than in the LT form, assuming comparable reorganization energies and not accounting for the influence of external experimental factors such as contact resistance variation.

We fabricated top contact, bottom gate thin film transistors (Supplementary Fig. 12a) to assess the effect of polymorphic transition on device performance. At room temperature, we measured apparent mobility of 1.9 cm^2^ V^−1^ s^−1^ in the saturation regime (*V*_d_ = −100 V) and 1.3 cm^2^ V^−1^ s^−1^ in the linear regime (*V*_d_ = −2 V). The transfer, output curves, and gate voltage-dependent mobility in the saturation regime are given in Fig. 6b, c, Supplementary Fig. 12b, and the linear regime results are shown in Supplementary Fig. 13. From temperature-dependent device measurements, we observed an inverse relationship between apparent mobility and temperature (Fig. 6d). The trend is a characteristic of band-like transport^57^, but no conclusions can be made as the intrinsic mobility may be obscured by the contact effect (Supplementary Fig. 12c). Irrespective of the charge transport mechanism, we note that decreased mobility at elevated temperatures can be linked to smaller transfer integrals along the herringbone direction in the HT form. Interestingly, we observed a steeper decay of mobility once the temperature surpassed the transition temperature near 340 K, supporting that the charge transport is indeed modulated by martensitic transition (Fig. 6d). This change in slope of mobility versus temperature curve agrees with a previous study by Jurchescu et al.^53^, which also concluded that the slope change resulted from polymorph transition.

In applying martensitic transition to functional materials, we are interested in testing whether cooperative transition can lead to function memory effect, specifically reversible change in electronic properties that are much more pronounced than simple temperature-dependent change. By cycling between LT and HT forms between 318 and 363 K, we observed excellent function memory effect in ditBu-BTBT devices with an approximate 217% change in charge mobility (Fig. 6e). To validate that the pronounced change observed is from the transition and not from the change in temperature, we calculated the average percent change of mobility within the same temperature range in other organic thin film and single-crystal transistors. The percent changes in other devices without any transition ranged from 11 to 60%, much lower than the change observed from ditBu-BTBT devices (Supplementary Table 5).

The thin film devices showed excellent recovery of performance and did not experience mechanical fracture thanks to the cooperative nature of the transition (Fig. 6f, Supplementary Fig. 14). The LT form showed higher mobility than the HT form, matching the trend from charge transfer integral calculations (Fig. 6a). However, the threshold voltage inevitably drifted to higher values with increasing number of thermal cycles (Supplementary Fig. 15a).

For TIPS-pentacene, we confirmed polymorphic transition in its thin film form based on in situ GIXD studies reported before^27^, with unit cell parameters of polymorphs I and IIb closely matching with the single-crystal diffraction results obtained at 300 and 400 K. Charge transport characteristics of TIPS-pentacene thin film transistors are shown in Supplementary Fig. 16. In an ideal case, we observed a sudden jump in the apparent mobility, accompanied by a gradual increase in the threshold voltage near the transition temperature measured from DSC and in situ GIXD^27^ (Supplementary Fig. 17). This observation is also in agreement with the significant increase in charge transfer integrals after transition^27^. However, majority of devices yielded unstable performance not as repeatable as the ditBu-BTBT device, likely due to much higher transition temperature and deteriorated OSC-metal contact for the TIPS-pentacene devices.

Similar to the case of ditBu-BTBT, we observed function memory effect in the TIPS-pentacene devices over multiple cycles, despite the higher temperature required to induce transition (Fig. 6g). The change in mobility between the two forms was approximately 117%, while prior temperature-dependent studies in absence of martensitic transition found approximately 40% change in mobility in the same temperature range of 110 K (Supplementary Table 6). We note that, in our case, large modulation of mobility higher than 100% can potentially be achieved over a much narrower temperature range of tens of degrees; this would not be possible without martensitic transition. The higher mobility in HT than LT form agrees with the calculated charge transfer integrals^27^. The threshold voltage shifted to higher values with increasing number of thermal cycles (Supplementary Fig. 15b). The obvious decrease in mobility over cycles results from the increase in contact resistance from prolonged exposure to high temperatures (Supplementary Fig. 18). Similar to ditBu-BTBT devices, these devices show excellent recovery of performance and preservation of thin film morphology before and after thermal cycles up to 423 K (Fig. 6h, Supplementary Fig. 19).

## Discussion

In conclusion, we propose the order-to-disorder transition of bulky side chains as the molecular origin of the cooperative transition behavior observed in two different high-performing organic electronics systems: ditBu-BTBT and TIPS-pentacene. We identify short-range repulsion between fast-rotating side chains as the main intermolecular force inducing cooperative movement. In TIPS-pentacene, cooperativity leads to a pronounced shape memory effect in millimeter-sized crystals. Cooperative transition induces function memory effect in both systems, showing significant reversible changes to the charge carrier mobility of thin film transistors. By tailoring the molecular structure to induce reversible function modulation, we are confronting a major challenge in materials design. Application of cooperative transition to organic electronics may find use in designing next-generation smart functional materials that exhibit shape memory effect, mechanical actuation, and electronic switching at the same time.

## Methods

### Synthesis

ditBu-BTBT was synthesized by Friedel–Crafts alkylation using the conditions of diacylation of BTBT^58^ with AlCl_3_ as Lewis acid. To a solution of BTBT (2.00 g, 8.32 mmol) in dry CH_2_Cl_2_ (150 mL) was added AlCl_3_ (3.99 g, 29.96 mmol) in one portion, at –20 °C and under an argon atmosphere. Then, the reaction mixture was cooled down to –78 °C. Next, *tert*-Butyl chloride (3.64 mL, 33.29 mmol) was then added dropwise in about 30 min. The reaction mixture was stirred at –78 °C for 2 h. The reaction was quenched by the addition of ice/water (100 mL). Volatiles were removed under reduced pressure. The formed precipitate was isolated by filtration, washed with water (3 × 50 mL) and methanol (3 × 50 mL), and dried. Further recrystallization in heptane afforded a white crystalline powder.^29^

### Single-crystal fabrication

Using a blend of 10 wt% polystyrene (MW 290k, Sigma Aldrich) and anhydrous tetralin (Sigma-Aldrich), 10 mg ml^−1^ of ditBu-BTBT was used as the stock solution. For TIPS-pentacene, 8 mg ml^−1^ solution of TIPS-pentacene (Sigma Aldrich) in anhydrous decane (Sigma-Aldrich) was used as the stock solution. To fabricate single crystals for in situ microscopy study, the stock solution was stirred and heated at 373 K (ditBu-BTBT) and 363 K (TIPS-pentacene) for 5 min, and 2 µL to 10 µL of the solution was dropcasted onto a clean or trichloro(octyl)silane modified silicon substrate (1 × 1 cm) at room temperature. The substrates were cleaned with toluene, acetone, and isopropanol, respectively. The drop was left in a hood under room temperature and moderate humidity to evaporate for at least 18 h. This procedure yielded single crystals with size ranging from 50 to 400 µm for ditbu-BTBT and 100 to 3 mm for TIPS-pentacene.

### Polarized optical microscopy

Single-crystal samples were placed in a heating chamber under a Nikon H550S with a high-speed camera (Infinity 1) and heating stage (Linksys32 temperature control). The heating chamber was capped with a sealable lid during heating and cooling cycles, and the rate was kept constant at 2 K min^−1^ (ditbu-BTBT) and 5 K min^−1^ (TIPS-pentacene). Time-lapse images were taken for all in situ experiments with various frame rates ranging from 5 to 40 fps. Image analysis was performed with softwares Nikon NIS Elements, Linksys32 data capture, and ImageJ.

### Differential scanning calorimetry

Thermal behavior of ditBu-BTBT single crystals was analyzed using a Perkin–Elmer Diamond 6 DSC instrument at 2 K min^−1^.

### Single-crystal characterization

Solution grown ditBu-BTBT and TIPS-pentacene single crystals were collected for single-crystal X-ray diffraction. Both were grown from a slow evaporation method in tetralin (3 mg mL^−1^ ditbu-BTBT) and acetone (1 mg mL^−1^ TIPS-pentacene). Diffraction data were collected on a Bruker D8 Venture equipped with a four-circle kappa diffractometer and Photon 100 detector. The Iμs microfocus Mo (*λ* = 0.71073 Å) source supplied the multi-mirror monochromated incident beam. The sample was mounted on a 0.3 mm loop with the minimal amount of Paratone-N oil. Data were collected as a series of φ and ω scans at the corresponding temperatures for the two systems. Data were integrated and filtered for statistical outliers using SAINT (Bruker, 2014) and then corrected for absorption by integration using SADABS v2014/4 (Bruker, 2014). The structure was phased by direct methods and refined using the software package SHELX-2014-3 (Sheldrick, 2008). The cif files were registered with the Cambridge Crystallographic Data Center (CCDC) as CCDC 1570908 (ditBu-BTBT at 298 K), 1570909 (ditBu-BTBT at 370 K), 1570910 (TIPS-pentacene at 300 K), and 1570911 (TIPS-pentacene at 400 K).

### NMR spectroscopy

All ^13^C variable temperature (VT) solid-state NMR experiments were performed at 17.6 T on a Varian NMR spectrometer system at the SCS NMR/EPR Facility of the University of Illinois at Urbana–Champaign, operating at resonance frequency of 188.4 MHz. The sample was finely ground and packed into 4 mm outer diameter zirconia rotors. A 4 mm Varian triple-resonance HXY T3 Narrow Bore magic angle spinning (MAS) probe was used for all MAS experiments under a spinning rate of 9750 Hz and two-pulse phase-modulation ^1^H decoupling. The spinning speed was chosen as such so that there would be minimum overlap between the spinning sidebands and the isotropic peaks. VT stack air flow set points used were 23, 37, 50, 57, 64, 71, 78, 85, and 91 °C, corresponding to sample temperatures of 30, 40, 50, 55, 60, 65, 70, 75, and 80 °C (±1°), as determined by calibration with ethylene glycol.

Experimental carbon chemical shift referencing, pulse calibration, and cross-polarization condition were done using powdered hexamethylbenzene, which has a chemical shift of 17.3 ppm (for the methyl peak) relative to the primary standard, tetramethylsilane at 0 ppm For cross-polarization MAS experiments, the ^1^H 90° pulse width was 6 µs, the contact time used was 3 ms, and the recycle delay was set to 2 s. Generally, 284–1024 scans were acquired with an acquisition time of 10 ms for each spectrum to get decent signal-to-noise ratio. Typical line broadening applied for all spectra was 10 Hz with 3 zero fills done to get 8192 data points in the frequency domain spectra.

### Raman spectroscopy

Raman spectra were taken from single crystals grown on Si substrates, the same samples made for in situ microscopy experiments. Raman confocal imaging microscope with a 532-nm laser (Laser Quantum Ventus 532 with max power 50 mW) and 50× long working distance objective lens (Horiba LabRAM HR 3D) equipped with Horiba Synapse back-illuminated deep-depletion CCD camera was used to collect spectrums. Using a 300 g mm^−1^ grating, scan exposure time of 1 s was used and five spectrums were averaged for each temperature. An optical density filter of OD = 0.6 was used (OD = log(power transmission factor) and no beam damage on the sample was observed,. The samples were collected in 5–10° increments from 298 to 373 K for both heating and cooling using a Linkam THMS600 heating stage. The heating and cooling rate was kept at 5 K min^−1^.

### Thin film transistor fabrication and characterization

A 10 mg mL^−1^ solution of ditBu-BTBT with 50 wt% polystyrene in tetralin and 8 mg mL^−1^ solution of TIPS-pentacene in toluene was used for meniscus-guided coating^30^ thin films onto a trichloro(phenyl)silane modified Si/SiO_2_ substrate with dielectric SiO_2_ of 300 nm thickness. Thickness of thus deposited films was measured using atomic force microscopy under the tapping mode using an Asylum Research Cypher instrument. Using a top contact bottom gate architecture, MoO_3_ (8 nm) and silver (35 nm) were thermally evaporated sequentially (Kurt J. Lesker Nano 36) onto shadow-masked ditBu-BTBT thin films as electrodes. The MoO_3_ layer was inserted to facilitate charge injection and alleviate the high contact resistance^29,59^, effectively reducing the threshold voltage from −83.4 to −68.7 V on average. Same evaporation method was applied to deposit gold (35 nm) electrodes for TIPS-pentacene based OFETs. All devices measured had a channel length of 73 and width of 4380 µm. Electronic performance was measured in a nitrogen atmosphere using a Keysight B1500A semiconductor parameter analyzer (Keysight Technologies). Field-effect mobility was calculated with standard equations for both the linear and the saturation regime, respectively (Eq. 1 and Eq. 2):

1 $$\mu _{{\mathrm{linear}}} = \frac{{\partial I_{{\mathrm{d}}}}}{{\partial V_{\mathrm{g}}}}\frac{L}{{WC_{\mathrm{i}}V_{{\mathrm{d}}}}}$$

2 $$\mu _{{\mathrm{sat}}} = \left( {\frac{{\partial \left( {I_{{\mathrm{d}}}} \right)^{0.5}}}{{\partial V_{\mathrm{g}}}}} \right)^2\frac{{2L}}{{WC_{\mathrm{i}}}}$$

where *L*, *W*, and *C*_*i*_ are channel length, channel width, and capacitance of the dielectric. *V*_d_ = −2 V was used for measurements in the linear regime and *V*_d_ = −100 V for the saturation regime for ditbu-BTBT, and *V*_d_ = −5 and *V*_d_ = −80 V for TIPS-pentacene. Temperature-dependent mobility measurements were conducted in the same semiconductor parameter analyzer with a temperature-controlled hot plate with a consistent heating and cooling rate of 5 K min^−1^ in a nitrogen atmosphere. At each temperature, the sample was given 1 min of stabilization time before measurement.

### Thin film structure characterizations

Thin film GIXD was performed at beam line 11–3 and 1–5 of Stanford Synchrotron Radiation Lightsource (SSRL) located at SLAC National Accelerator Laboratory. The X-ray beam energy was 12.73 keV and the incidence angle was 0.12°. X-ray diffraction images were collected on a 2D image plate (MAR345, 2300 × 2300 pixels, effective pixel size 150 µm). The distance between the image plate and sample stage was 400 mm. A helium chamber was used to minimize noise and a heated sample stage was used for detecting polymorphic transitions during sample annealing. ‘2D powder’ samples were fabricated to acquire the maximum number of peaks, and in a heat chamber the temperature was changed to observe peak shifts. The data analysis was performed using the WxDiff software and customized unit cell indexing algorithm.

### Computational methods

DFT calculations were carried out using (if not specified otherwise) CASTEP^60^ (Accelrys, San Diego, CA) Academic Release version 8.0, which implements DFT within a generalized gradient approximation with semi-empirical dispersion correction scheme TS^61^ and the plane-wave pseudopotential approach. All calculations used the Perdew−Burke−Ernzerhof (PBE) exchange-correlation functional^62^ with ultrasoft pseudopotentials^63^ and a basis set cutoff energy of 800 eV together with integrals taken over the Brillouin zone by using a Monkhorst–Pack grid of minimum sample spacing 0.1 × 2π Å^−1^. The crystal structures of LT and HT phases were determined from single-crystal X-ray diffraction in this study. Transfer integral calculations of LT and HT were performed starting from crystal structures calculated from single-crystal X-ray diffraction. During the PES scan calculations, corresponding forces, energies, and displacements at every step of rotation angle variation were converged to better than 0.01 eV Å^−1^, 0.00001 eV, and 0.001 Å, respectively.

Calculations of transfer integrals were performed on the isolated π–π and herringbone-like molecular pairs extracted from respective periodic crystal structures of LT and HT, in which the positions of all atoms in the unit cell were preserved as they appear in the crystal structure. This so-called ‘fragment’ approach allows to reliably calculate corresponding transfer integrals at the DFT level (PBE/DZ) with the Amsterdam Density Functional (ADF) package^64^ as described elsewhere^65,66^.

Classical MD calculations, for profiling ditbu groups rotation during thermal annealing, were done within the Materials Studio Package 6.0 (Materials Studio-v6.0.0, Accelrys Software Inc., San Diego, CA, 2011) using an general version of the Dreiding force field^67^ together with electrostatic potential charges, which were obtained via charge analysis using the Gaussian09 package^68^ with the PBE functional and the 6-311G(d,p) basis set.

Classical MD (NPT ensemble) simulations were performed imposing a supercell of 2 × 4 × 4 in size in order to allow symmetry and replica independent motion of 64 molecules of ditBu-BTBT in a unit cell. The unit cell and atomic coordinates were initially taken from the available X-ray crystal structure of LT. In order to allow for all local degrees of freedom to relax prior to performing a production MD run at elevated temperatures, the MD simulation was divided into two stages: starting with an initial thermalization MD run of 100 ps and followed by a production MD run (annealing and cooling) of in total 400 ps.

Correlations in the torsional dynamics of neighboring ditBu groups have been quantified by computing the average correlations coefficient of the time series of ditBu-BTBT torsional angles *ϕ* extracted from the MD trajectory. For every pair of neighboring angles *i*–*j* we have computed the linear correlation coefficient.

3 $$R_{{ij}}\left( T \right) = \left| {\frac{{{\rm cov}\left( {\phi _{i},\phi _{j}} \right)}}{{\sigma _{i}\sigma _{j}}}} \right|$$

where the covariance (cov) and the standard deviations (*σ*) are computed for time series of 20 ps of lengths centered on a given *T*. The time window of 20 ps approximately corresponds to the coherence time of the rotational motion, and different values in a reasonable range (10–30 ps) give comparable results. The absolute value in Eq. 3 comes from the fact that we are interested in correlations in a broad sense, without distinguishing between positive (*R* > 0) and negative correlations (*R* < 0) that were both observed in our dynamics. The mean correlation coefficient *R* is then calculated with an average over all the pairs of neighboring torsions, defined as those with distance lower than 7 Å. The result is weakly sensitive to the choice of the cutoff distance.

### Data availability

The authors declare that the main data supporting the findings of this study are available within the paper and the Supplementary Information. Data are available from the corresponding author upon request.

## Electronic supplementary material

Supplementary Information

Description of Additional Supplementary Files

Supplementary Movie 1

Supplementary Movie 2

Supplementary Movie 3

Supplementary Movie 4

Supplementary Movie 5
