# Supplementary material for: Rotator side chains trigger cooperative transition for shape and function memory effect in organic semiconductors
Source: Nat Commun. 2018 Jan 18;9:278. doi: 10.1038/s41467-017-02607-9 (PMC5773606; doi:10.1038/s41467-017-02607-9)
Supplement: Supplementary file 1 — Supplementary Information [file 41467_2017_2607_MOESM1_ESM.pdf]

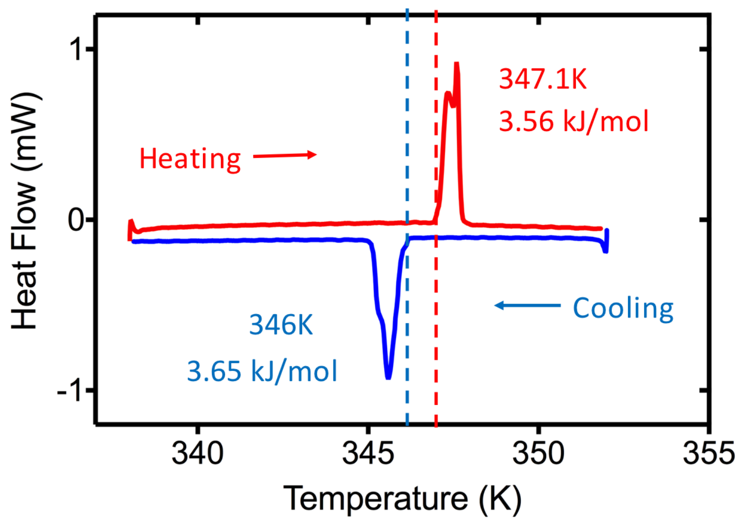

Supplementary Figure 1 | Differential scanning calorimetry curve of ditBu-BTBT single crystal. A single crystal sample was subject to 2 $Kmin^{-1}$ heating and cooling rate for DSC analysis. Heating and cooling transition temperatures and enthalpy values are provided.

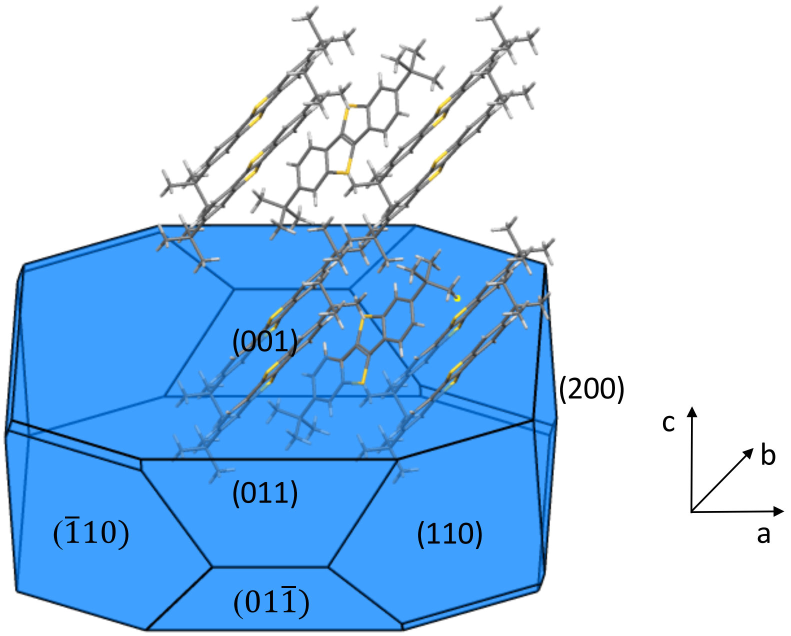

Supplementary Figure 2 | BFDH calculation from ditBu-BTBT single crystal structure data. Morphology determined from Mercury software. Main (hkl) planes noted on the plane. The largest (001) plane is assumed as the surface observed from in situ POM and hence the molecular packing is estimated as depicted in Fig.1b.

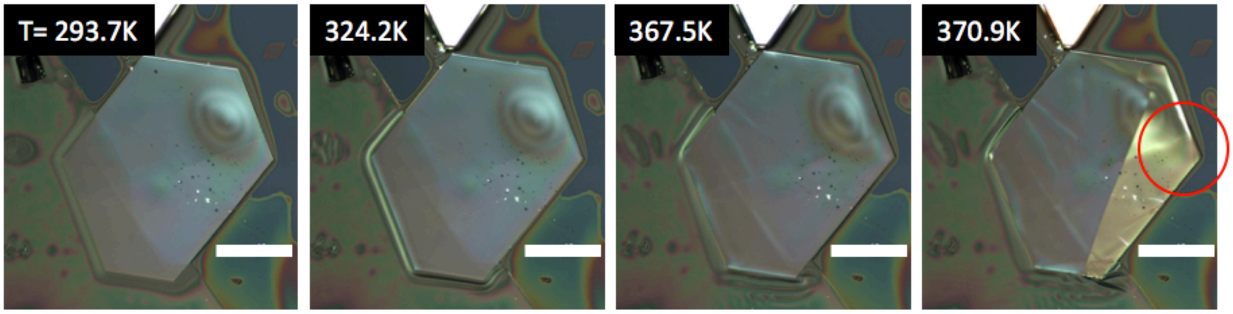

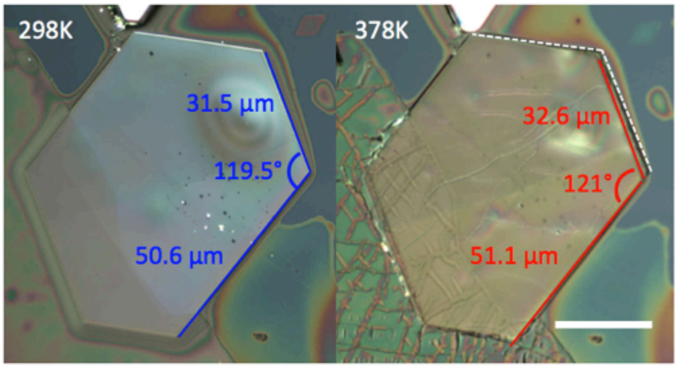

Supplementary Figure 3 | Shape memory effect in a ditBu-BTBT single crystal. Compared to TIPS-pentacene, a much smaller shape memory effect is observed from the length and the angle of the crystal after transition. However, we can see that the crystal remains stationary before transition (293.7 to 367.5K), but upon the start of transition at 370.9K, the crystal shows shape change. The bottom images show the LT and HT forms, with the original position of the LT crystal in white dashed lines. The slight movement of the crystal from transition induces mechanical deformation to the crystal underneath, causing it to crack. All scale bars correspond to 25 µm.

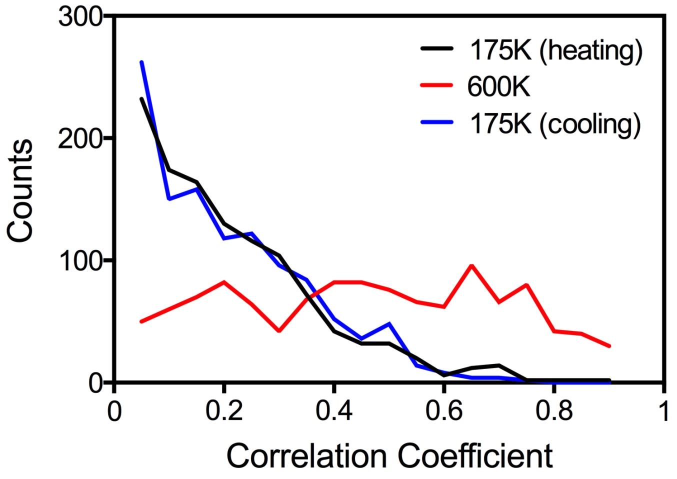

Supplementary Figure 4 | Distribution of the correlation coefficient values at 175K and 600K. The values were calculated for all the pairs of neighboring ditBu groups at 175K upon heating, 600K, and 175K upon cooling.

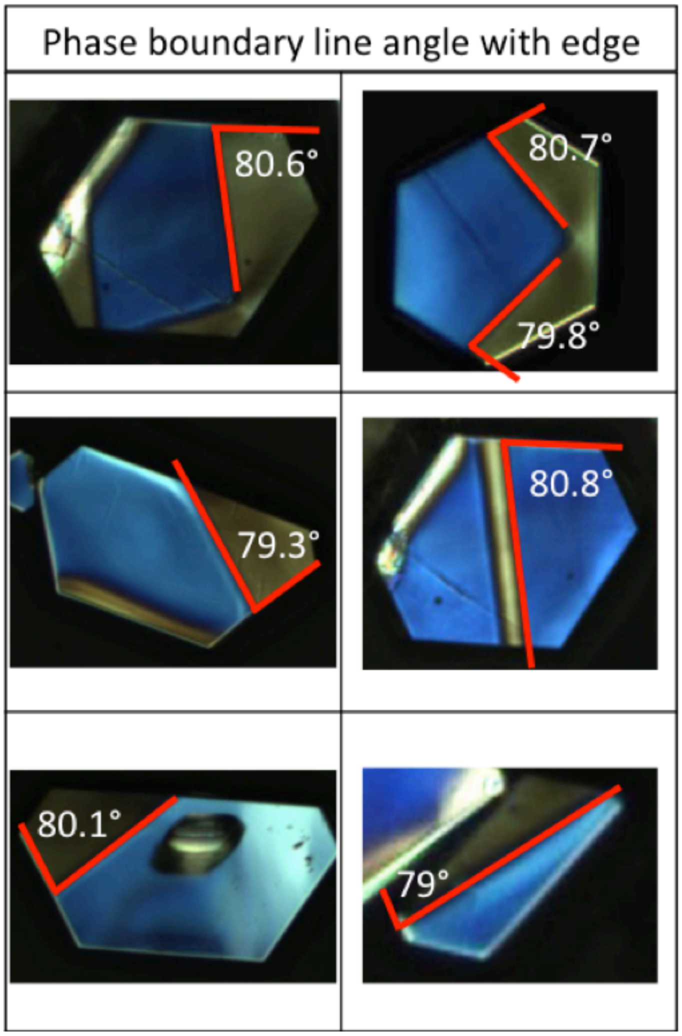

Supplementary Figure 5 | Similar angles between the phase boundary line and crystal edge. A similar angle is measured throughout all single crystal samples, indicating that a common phase boundary line appears for all cooperative transition. Brightness, contrast, and sharpness of the images were enhanced for better visualization.

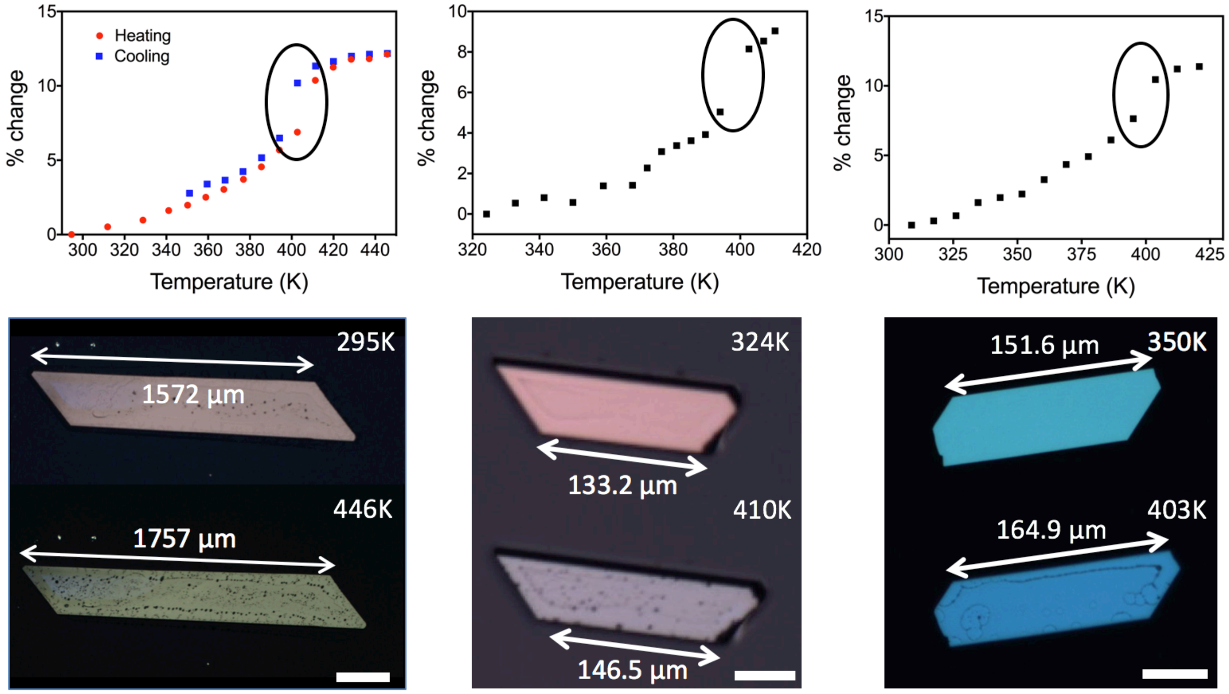

Supplementary Figure 6 | Shape memory effect in TIPS-pentacene single crystals. Plot of percent change of a crystal edge during a thermal cycle. A pronounced reversible shape memory effect is observed, showing up to a 12% increase after transition. A sudden change in length was observed near 400K, consistent with the solid-state polymorphic transition occurring at 395K identified by bulk DSC. Change in crystal length for heating and cooling in the first case shows a clear hysteresis. This agrees with both microscopy and DSC studies, indicating that the transition is first order, same as in the case of ditBu-BTBT. The process is shown in Supplementary Movie 3. Scale bars correspond to 300 µm, 50 µm, and 50 µm from left to right, respectively.

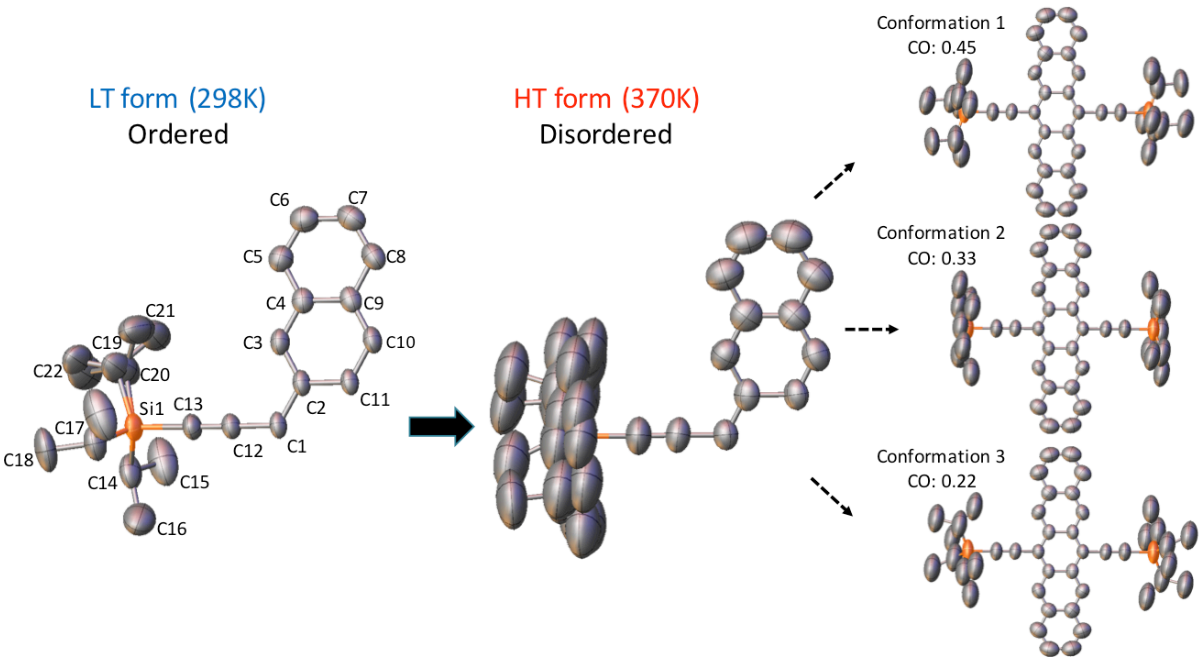

Supplementary Figure 7 | Single crystal X-ray diffraction results of the order to disorder transition of the side chains. The figure shows thermal ellipsoid representation with 50% probability. The highly static positions of the side chains in the LT form transitions into a highly disordered state with three distinct conformations measured from SCXRD. Chemical occupancy (CO) of each of the disordered forms are indicated.

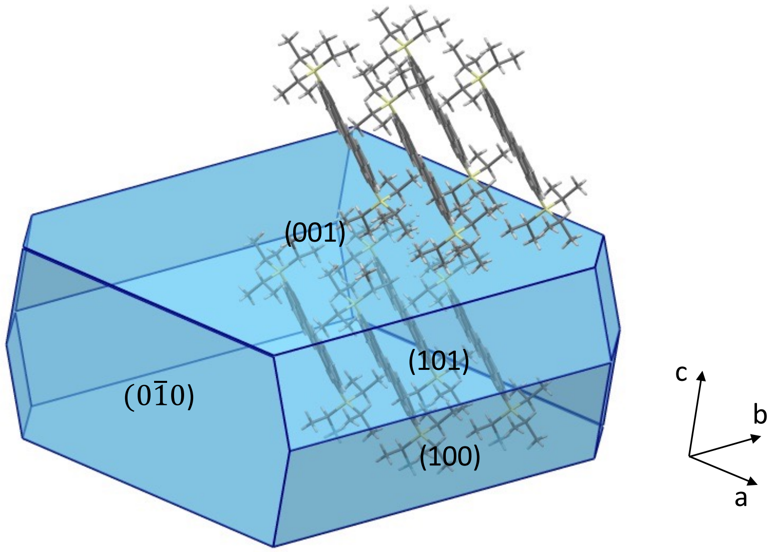

Supplementary Figure 8 | BFDH calculation from TIPS-pentacene single crystal structure data. Morphology determined from Mercury software. Main (hkl) planes noted on the plane. The largest (001) plane is assumed as the crystal surface observed from in situ POM.

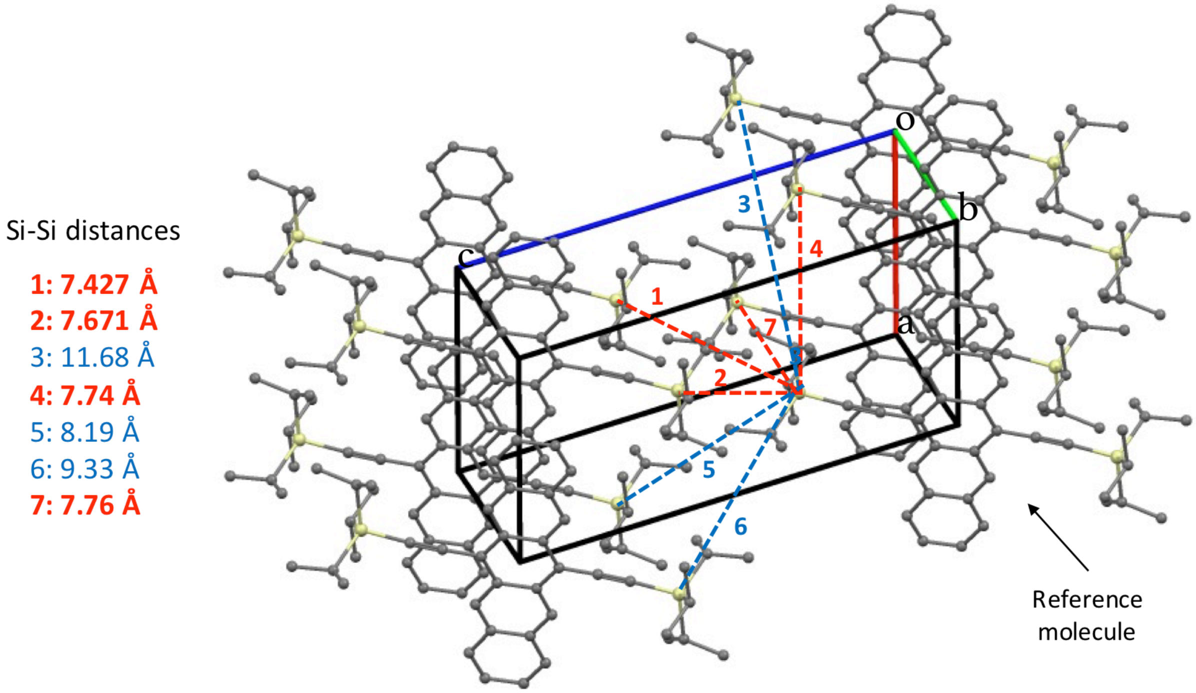

Supplementary Figure 9 | Measurement of Si-Si intermolecular distances of neighboring TIPS-pentacene molecules. Four molecular pairs 1,2,4, and 7 have relatively short Si-Si distances, indicating that they may experience stronger repulsive force when side chains start rotating. Note that the influence of the short range repulsive force occurs in all three directions and that the rotating side chains along the *a* axis may explain the shape memory effect along the same axis.

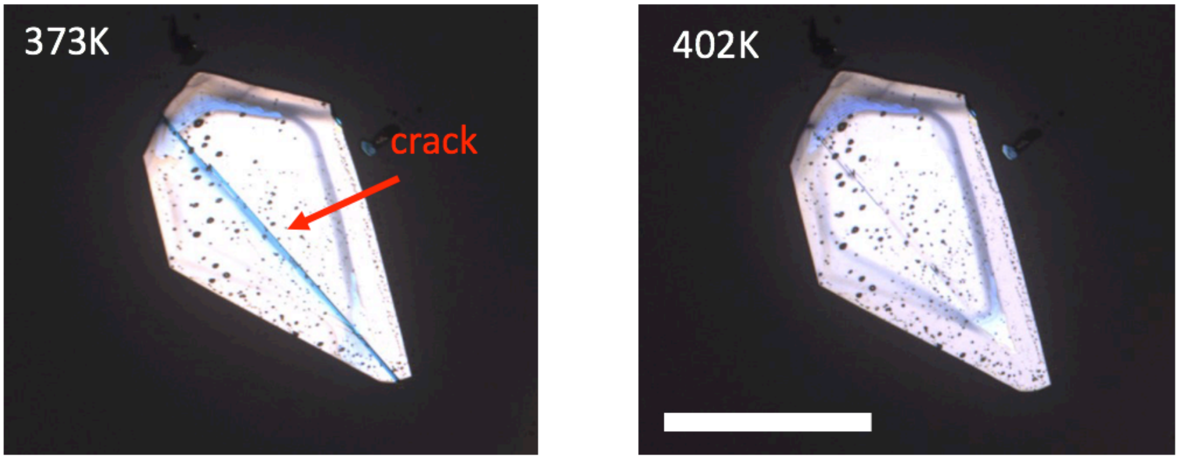

Supplementary Figure 10 | Self-healing effect in TIPS-pentacene single crystal. The sample was continuously heated up to 433K and cooled down to 373K until a crack developed upon its 8th cooling cycle. The POM images above are taken on the 9th heating cycle. The crack healed itself as the crystal passed the transition temperature for cooperative transition (see Supplementary Movie 4). Scale bar corresponds to 500 μm.

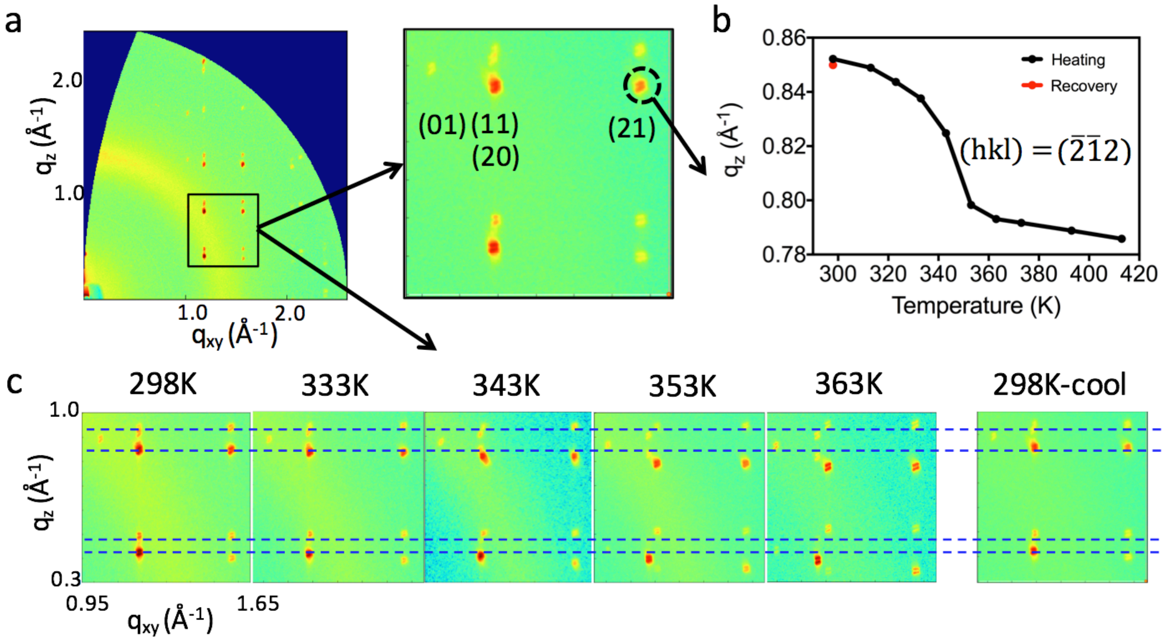

Supplementary Figure 11 | In situ GIXD of ditBu-BTBT thin films. (a) Molecular characterization in thin films of ditBu-BTBT using variable temperature GIXD. Peak shift is prominent as the stage is heated up to 363K. (b) The q_z peak value of a high-intensity diffraction peak ($\bar{2}\,\bar{1}\,2$) during heating, along with the recovered value in red after cooling down to 298K. (c) The polymorphic transition started at 343K, compared to 345K for single crystals upon heating. This out-of-plane shift agreed with the observed out-of-plane tilt in the unit cell. When the sample was cooled back to room temperature, the q_z shifted back to its initial value, proving the reversibility of transition.

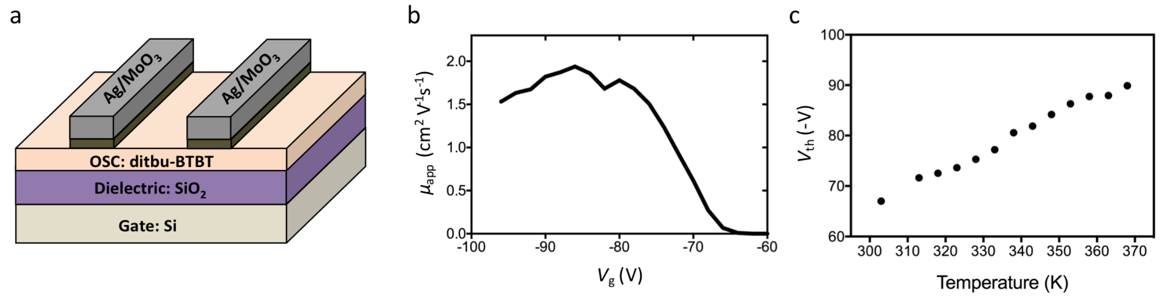

Supplementary Figure 12 | Thin film transistor configuration and charge transport characteristics in ditBu-BTBT. (a) Top contact bottom gate thin film transistor configuration. Same configuration with gold as the electrode was used for TIPS-pentacene thin film transistors. (b) Saturation regime (V_d = -100V) apparent mobility as a function of gate voltage at room temperature. The plot displays a plateau region for higher voltage, indicating a relatively stable performance. (c) Increase in contact resistance of device manifested by the change in threshold voltage upon heating.

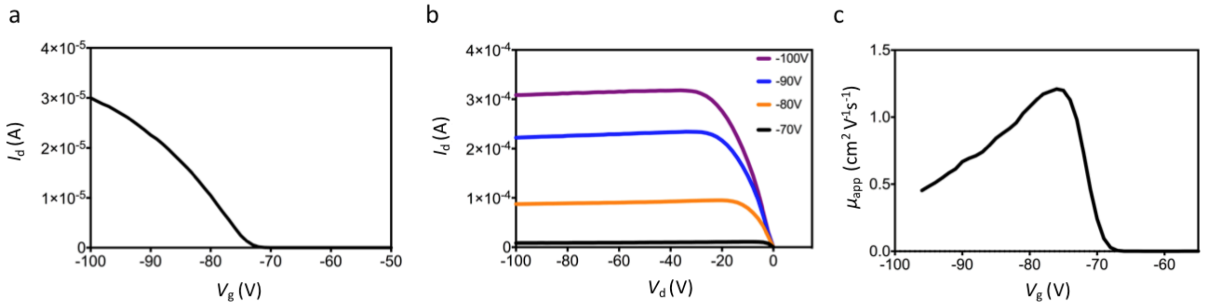

Supplementary Figure 13 | Linear regime electronic characteristics in ditBu-BTBT thin film transistors. (a) Transfer curve (V_d = -2V), (b) Output curve and (c) Gate voltage dependent charge mobility. Note that compared to the saturation regime, there is a significant decrease in mobility at the higher gate voltage region.

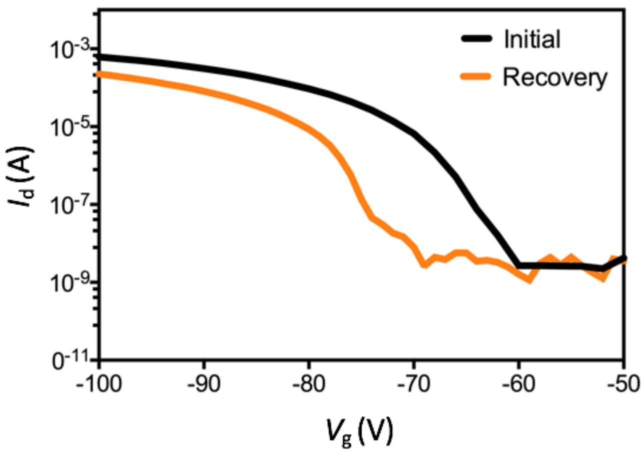

Supplementary Figure 14 | Recovery of ditBu-BTBT thin film devices. Transfer curves initially and after a thermal cycle.

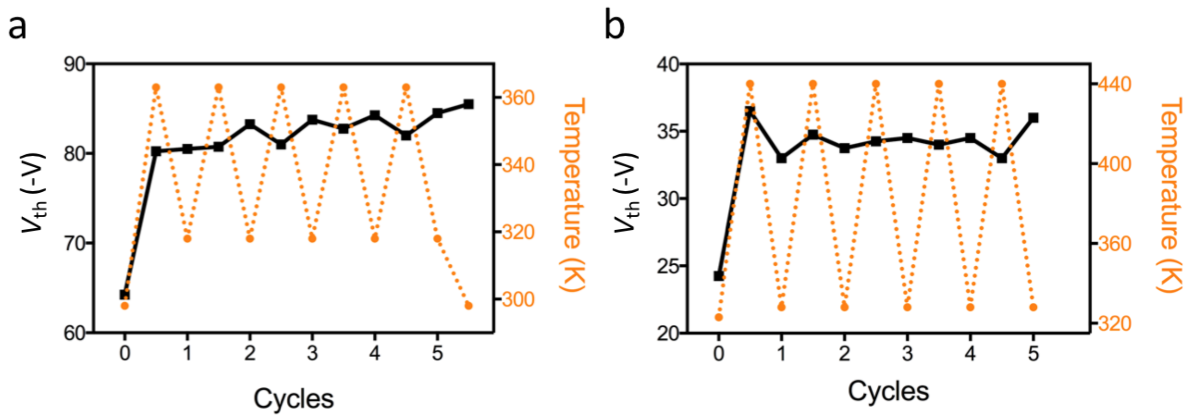

Supplementary Figure 15 | Change in threshold voltage for multiple thermal cycles in thin film transistors. (a) Change in threshold voltage (left axis,black) and temperature (right axis,orange) in ditBu-BTBT thin film transistors. (b) Change in threshold voltage (left axis,black) and temperature (right axis,orange) in TIPS-pentacene thin film transistors.

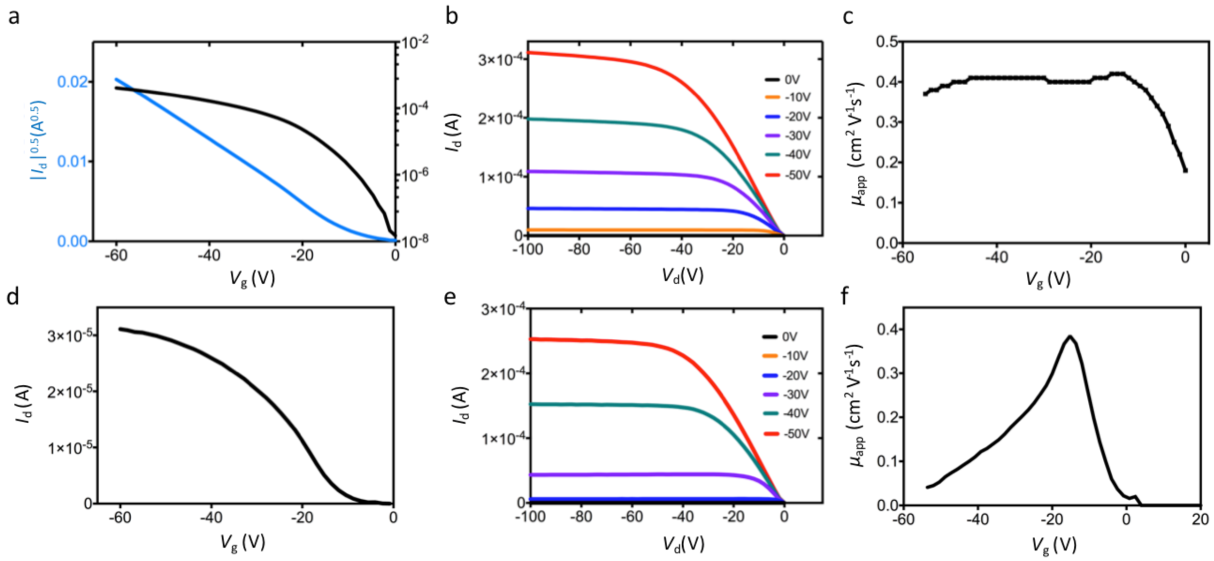

Supplementary Figure 16 | Saturation and linear regime electronic characteristics in TIPS-pentacene thin film transistors. (a) Saturation regime transfer curve (V_d = -80V). Note that TIPS-pentacene devices have very low threshold voltage. (b) Output curve (c) Gate voltage dependent apparent charge mobility. The plateau is observed almost throughout the whole gate voltage range. (d) Linear regime transfer curve (V_d = -5V) (e) Output curve (f) Gate voltage dependent apparent charge mobility. There is a sharp decrease of mobility for higher gate voltage in the linear regime.

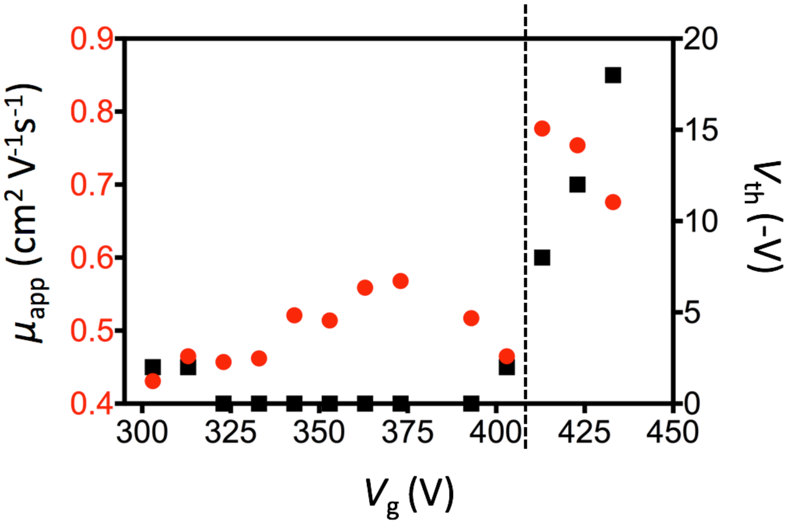

Supplementary Figure 17 | Temperature dependent apparent mobility and threshold voltage in TIPS-pentacene thin film transistors. The dashed line indicates the polymorphic transition temperature observed from in situ GIXD.

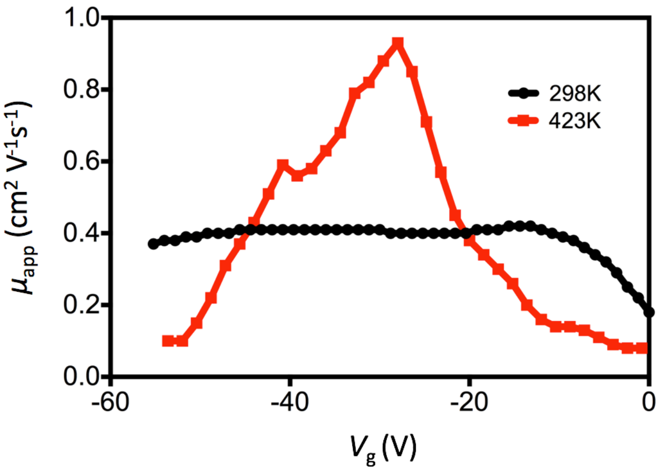

Supplementary Figure 18 | Instability of TIPS-pentacene thin film devices at high temperature. Gate voltage dependent charge mobility for room temperature (black) and high temperature after transition (red) are plotted. When the device is heated to high temperature and experience martensitic transition, it becomes unstable, shown by the significant decrease in the mobility for higher gate voltage. Thus the higher apparent mobility observed for the high temperature form cannot be trusted and we could not determine a clear dependence of the device on temperature.

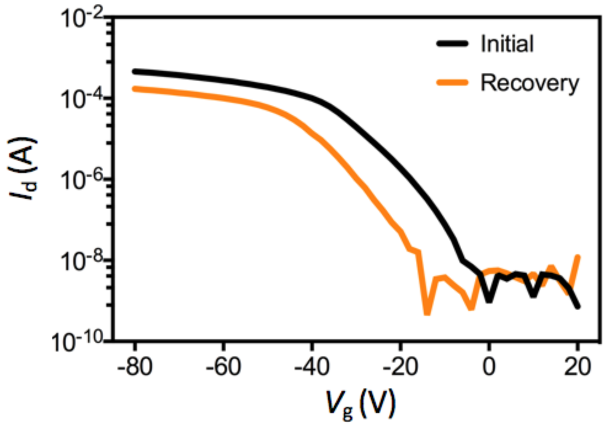

Supplementary Figure 19 | Recovery of performance in TIPS-pentacene thin film devices after thermal cycling. Transfer curves of the device initially and after a thermal cycle.

Crystal	T1H	T1C	T2H	T2C	T3H	T3C	T4H	T4C	T5H	T5C	T6H	T6C	T7H	T7C	T8H	T8C	T9H	T9C
1	343.7	329.8	343.5	330.0	343.2	331.2	341.8	330.1	341.5	331.5	340.9	335.8	340.8	336.8	342.4	336.8	341.3	338.1
2	344.6	329.7	345.3	332.4	344.6	331.1	343.7	329.7	344.0	332.0	343.3	332.7	342.5	330.5	342.7	330.0	342.5	333.0
3	344.5	329.9	344.3	334.0	343.7	334.1	342.0	332.6	342.4	333.0	341.7	332.6	342.1		341.8	333.1	342.4	333.2
4	344.1	328.5	344.9	329.4	343.0	333.5	341.7	336.7	340.5	333.7	340.5	334.8	340.2		340.5	335.0	340.5	333.4
5	344.4	329.9	344.9	334.0	344.3	334.1	343.0	331.7	342.5	330.9	341.7	332.6	341.9		341.8	335.4	341.3	331.8
6	343.7	329.0	342.6	331.5	342.5	331.0	341.0	330.0	341.2	333.1	341.0	334.0	341.0		341.1	333.0	341.0	330.5
7	344.3	335.1	344.3	332.3	343.8	332.7	342.4	330.2	342.4	335.5	341.3	332.4	341.5		341.2	335.4	341.2	333.1
8	344.1	329.8	343.8	330.2	343.7	331.7	341.8	331.4	342.6	334.3	341.7	331.7	341.7		341.7	331.9	341.3	332.7
9	344.0	327.5	343.3	327.6	342.9	329.1	342.4	328.6	342.4	329.0	342.4	329.9	342.6	335.6	342.1	330.5		
10	344.7	334.5	343.8	335.2	343.7	335.7	343.4	332.9	343.7	334.3	343.7	337.4	343.7	336.2	343.3	337.6		
11					343.7	330.7	343.3	330.9	343.1	332.2	342.9	331.7	342.8	331.8	342.4	333.0		
12	344.7	327.8	344.5	327.4	344.3	327.2	344.0	328.6	343.9	329.4	343.7	331.5	343.0	331.7	343.7	331.5		
13	344.1	329.1	344.3	328.5	344.1	327.9	344.2	331.0	344.3	331.1	344.2	334.9	344.1	333.1	343.8	332.4		
14	341.8	331.8	342.0	334.4	341.8	335.5	341.4	333.9	341.4	334.5	341.3	338.1	341.5	336.0	341.2	335.3		
15	343.5	337.2	341.7	337.1	341.3	336.8	341.6	337.3	341.2	337.2								
16	344.6	328.0			343.0	333.3												
17	344.4	328.5	342.7	329.8	342.6	333.3	342.4	330.7	342.2	330.5	342.2	333.0						
18	343.5	327.1	342.8	334.7	342.6	331.4	342.7	333.6	342.8	332.9	342.4	331.7						
19	344.4	331.7	342.1	334.8	342.4	334.6	342.4	335.5	341.8	335.9	341.8	334.9						
20	344.4	333.1	342.7	333.0	342.4	333.4	342.3	333.0	342.4	333.6	342.4	333.8						
21	344.0	332.6	342.6	333.5	342.3	334.3	342.2	334.5	342.2	336.6	342.0	336.9						
22	343.9	332.5	343.1	333.4	343.0	334.2	343.0	334.3	342.2	334.3	342.4	334.1						
23	343.9	329.0	343.6	330.1	343.7	333.2	343.0	332.4	341.8	335.2	342.6	333.3						
24	344.1	329.2	343.3	330.1	342.9	333.2	341.8	332.4	341.8	334.2	342.6	333.3						
25	343.6	325.2	343.7	333.7	342.5	329.3	342.2	333.5	342.1	329.8	343.1	332.7						
26	350.2	334.9	348.5	341.4	348.1	340.5	348.0	342.4	347.9	341.8								
27	350.2	336.8	347.4	335.1	348.1	340.1	347.3	341.2	347.6	340.8								
28	350.0	330.6	349.7	335.6	348.2	335.1	347.5	340.8	347.4	342.4	347.4	342.6						
29	349.5	337.4	348.5	339.3	347.7	339.9	347.6	339.3	348.1	340.6	348.3	340.2						
30	349.0	334.0	348.1	335.3	347.8	333.6	347.5	335.3	347.4	335.8	347.5	334.9						

Supplementary Table 1 | Measured transition temperatures for all crystal samples over multiple cycles. The H and C in the first row refer to heating and cooling transition temperatures, respectively. All temperature units are in Kelvin. Note that some transition temperatures were not obtained due to technical difficulties. Total of 30 single crystals were measured. Note that the average transition temperatures of heating and cooling given in the manuscript are averages of the first cycle values.

	ditBu-BTBT: LT form		ditBu-BTBT: HT Form	
Empirical formula	$C_{22}H_{24}S_2$		$C_{22}H_{24}S_2$	
Formula weight	352.53		352.53	
Temperature / K	298(2)		370(2)	
Wavelength / Å	0.71073		0.71073	
Crystal system	Monoclinic		Monoclinic	
Space group	P21/c		P21/c	
Unit cell dimensions	a = 14.1772(6) Å<br>b = 6.1547(3) Å<br>c = 10.6485(5) Å	α= 90°.<br>β= 92.2098(16)°.<br>γ= 90°.	a = 14.6338(12) Å<br>b = 6.3264(6) Å<br>c = 10.4185(9) Å	α= 90°.<br>β= 93.755(3)°.<br>γ= 90°.
Volume / Å^3	928.46(7)		962.47(15)	
Z	2		2	
$\rho_{calculated}$ / (Mg/m^3)	1.261		1.216	
Absorption coefficient / mm^{-1}	0.287		0.277	
F(000)	376		376	
Crystal size / mm^3	0.433 x 0.222 x 0.054		0.433 x 0.222 x 0.054	
Theta range for data collection	2.876 to 25.368°.		2.790 to 25.492°.	
Index ranges	-17<=h<=17<br>-7<=k<=6<br>-12<=l<=12		-17<=h<=17<br>-7<=k<=6<br>-12<=l<=12	
Reflections collected	10961		11043	
Independent reflections	1707 [R(int) = 0.0262]		1786 [R(int) = 0.0456]	
Completeness to theta = 25.242°	99.90%		99.80%	
Absorption correction	Integration		Integration	
Max. and min. transmission	0.7452 and 0.7022		0.7452 and 0.6638	
Refinement method	Full-matrix least-squares on F2		Full-matrix least-squares on F2	
Data / restraints / parameters	1707 / 103 / 153		1786 / 280 / 194	
GoF	1.103		1.072	
Final R indices [I>2sigma(I)]	R1 = 0.0339<br>wR2 = 0.0899		R1 = 0.0448<br>wR2 = 0.1198	
R indices (all data)	R1 = 0.0376<br>wR2 = 0.0931		R1 = 0.0595<br>wR2 = 0.1316	
Extinction coefficient	0.138(9)		0.160(13)	

Supplementary Table 2 | Crystallographic data and refinement statistics for ditBu-BTBT at 298K and 370K.

Source	Chung	Chung	Chung	Ref. 1	Ref. 1	Ref. 1	Dudenko	Dudenko
Sample	SC	SC	SC	SC	Thin film (40 nm)	Thin film (40 nm)	MD simulation	MD simulation
T (K)	298	370	400	298	298	353	298	370
a (Å)	10.65	10.42	10.43	13.97	10.61	10.39	10.5	10.0
b (Å)	6.15	6.33	6.36	6.10	6.134	6.25	6.0	6.0
c (Å)	14.18	14.63	14.67	10.57	14.07	14.54	13.9	14.7
α (Å)	90	90	90	90	90	90	90	90
β (Å)	92.2	93.8	93.9	92.67	92.3	93.7	91.7	93.3
γ (Å)	90	90	90	90	90	90	90	90
Volume ($Å^3$)	928.5	962.5	969.4	899.9	915.1	941	870	878

Supplementary Table 3 | Unit cell parameters from single crystal X-ray diffraction and thin film grazing incidence X-ray diffraction for ditBu-BTBT. Unit cell parameters from single crystal X-ray diffraction, refined calculations from combination of Synchrotron GIXD and refinement software for thin film[1], and CASTEP estimations from MD simulations. The details of structure refinement method are given in reference 1.

	TIPS-pentacene: LT form		TIPS-pentacene: HT Form	
Empirical formula	$C_{44}H_{54}Si_2$		$C_{44}H_{54}Si_2$	
Formula weight	639.05		639.05	
Temperature / K	300(2)		400(2)	
Wavelength / Å	0.71073		0.71073	
Crystal system	Triclinic		Triclinic	
Space group	P-1		P-1	
Unit cell dimensions	a = 7.7440(7) Å	α= 88.536(4)°	a = 7.600(8) Å	α = 78.22(3)°
	b = 7.7583(8) Å	β = 77.873(3)°	b = 8.578(9) Å	β = 86.71(3)°
	c = 16.9549(17) Å	γ = 82.202(4)°	c = 17.231(17) Å	γ = 72.10(3)°
Volume / Å^3	986.71(17) Å3		1046.5(19) Å3	
Z	1		1	
$\rho_{calculated}$ / (Mg/m^3)	1.075		1.014	
Absorption coefficient / mm^{-1}	0.118		0.111	
F(000)	346		346	
Crystal size / mm^3	1.056 x 0.243 x 0.104		1.056 x 0.243 x 0.104	
Theta range for data collection	2.457 to 25.500°		2.415 to 17.997°	
Index ranges	-9<=h<=9, -9<=k<=9, -20<=l<=20		-6<=h<=6, -7<=k<=7, -14<=l<=14	
Reflections collected	15410		5599	
Independent reflections	3665 [R(int) = 0.0283]		1426 [R(int) = 0.0505]	
Completeness to theta = 25.242°	99.90%		98.60%	
Absorption correction	Semi-empirical from equivalents		Semi-empirical from equivalents	
Max. and min. transmission	0.7452 and 0.7022		0.7445 and 0.5708	
Refinement method	Full-matrix least-squares on F2		Full-matrix least-squares on F2	
Data / restraints / parameters	3665 / 92 / 245		1426 / 707 / 347	
GoF	1.114		2.773	
Final R indices [I>2sigma(I)]	R1 = 0.0526, wR2 = 0.1414		R1 = 0.1226, wR2 = 0.3396	
R indices (all data)	R1 = 0.0661, wR2 = 0.1526		R1 = 0.1494, wR2 = 0.3560	
Extinction coefficient	0.056(7)		0.066(10)	

Supplementary Table 4 | Crystallographic data and refinement statistics for TIPS-pentacene at 300K and 400K.

System	T range	Mobility change (%)	Reference
**ditBu-BTBT TFT**	**55 (363-318K)**	**217**	**Our work**
TIPS-pentacene TFT	55 (300-245K)	14.8	Sakanoue *et al.*[2]
Polymer (P8T2Z-C12) TFT	40 (330-290K)	59	Lee *et al.*[3]
Rubrene SCFET	50 (300-250K)	11.3	Okada *et al.*[4]
	50 (300-250K)	25	Podzorov *et al.*[5]

Supplementary Table 5 | Comparison of the change in mobility in ditBu-BTBT with other transistors. TFT refers to thin film transistors, SCFET refers to single crystal transistors.

System	T range	Mobility change (%)	Reference
**TIPS-pentacene TFT**	**112 (328-440K)**	**117**	**Our work**
TIPS-pentacene TFT	110 (300-190K)	40.7	Sakanoue *et al.*[2]
Rubrene SCFET	100 (300-200K)	40	Podzorov *et al.*[5]

Supplementary Table 6 | Comparison of the change in mobility in TIPS-pentacene with other transistors. TFT refers to thin film transistors, SCFET refers to single crystal transistors.

Supplementary References

1 Schweicher, G. *et al.* Bulky End-Capped [1]Benzothieno[3,2-b]benzothiophenes: Reaching High-Mobility Organic Semiconductors by Fine Tuning of the Crystalline Solid-State Order. *Advanced Materials* **27**, 3066-3072 (2015).

2 Sakanoue, T. & Sirringhaus, H. Band-like temperature dependence of mobility in a solution-processed organic semiconductor. *Nat Mater* **9**, 736-740 (2010).

3 Lee, J. *et al.* Thin Films of Highly Planar Semiconductor Polymers Exhibiting Band-like Transport at Room Temperature. *Journal of the American Chemical Society* **137**, 7990-7993 (2015).

4 Okada, Y., Sakai, K., Uemura, T., Nakazawa, Y. & Takeya, J. Charge transport and Hall effect in rubrene single-crystal transistors under high pressure. *Phys Rev B* **84** (2011).

5 Podzorov, V. *et al.* Intrinsic charge transport on the surface of organic semiconductors. *Physical Review Letters* **93** (2004).
