# Supplementary material for: Rotator side chains trigger cooperative transition for shape and function memory effect in organic semiconductors
Source: Nat Commun. 2018 Jan 18;9:278. doi: 10.1038/s41467-017-02607-9 (PMC5773606; doi:10.1038/s41467-017-02607-9)
Supplement: Supplementary file 2 — Description of Additional Supplementary Files [file 41467_2017_2607_MOESM2_ESM.pdf]

Description of Additional Supplementary Files

File Name: Supplementary Movie 1
Description: ditBu-BTBT single crystal showing Type I martensitic transition under in situ POM upon heating. The video shows selected frames from the first heating cycle that show the complete transition process of the crystal on the upper left corner. It is the same crystal sample as the one shown in Fig.1a.

File Name: Supplementary Movie 2
Description: ditBu-BTBT single crystal showing Type I martensitic transition under in situ POM upon cooling

File Name: Supplementary Movie 3
Description: ditBu-BTBT single crystal showing Type II martensitic transition under in situ POM. Same crystal sample as the one shown in Fig.1a. Brightness settings altered for better visual presentation.

File Name: Supplementary Movie 4
Description: TIPS-pentacene single crystal showing shape memory effect under in situ POM

File Name: Supplementary Movie 5
Description: TIPS-pentacene single crystal showing self-healing under in situ POM. Repeated thermal cycles cracked the sample in the 8th cooling cycle, and for 6 heating cycles starting from the 9th cycle, the crack "healed" as transition was taking place. As seen from the video, the cooperative nature of martensitic transition allows the recovery of the crack as the sample reaches near the transition temperature.
